# Type I and Type III Interferons Display Different Dependency on Mitogen-Activated Protein Kinases to Mount an Antiviral State in the Human Gut

**DOI:** 10.3389/fimmu.2017.00459

**Published:** 2017-04-21

**Authors:** Kalliopi Pervolaraki, Megan L. Stanifer, Stephanie Münchau, Lynnsey A. Renn, Dorothee Albrecht, Stefan Kurzhals, Elena Senís, Dirk Grimm, Jutta Schröder-Braunstein, Ronald L. Rabin, Steeve Boulant

**Affiliations:** ^1^Schaller Research Group at CellNetworks, Department of Infectious Diseases, Virology, Heidelberg University Hospital, Heidelberg, Germany; ^2^Research Group “Cellular Polarity and Viral Infection” (F140), German Cancer Research Center (DKFZ), Heidelberg, Germany; ^3^Center for Biologics Evaluation and Research, US Food and Drug Administration, Silver Spring, MD, USA; ^4^Institute of Immunology, Heidelberg University Hospital, Heidelberg, Germany; ^5^Department of Infectious Diseases, Virology, BioQuant, Heidelberg University Hospital, Heidelberg, Germany

**Keywords:** interferon-lambda, interferon-β, intestinal epithelial cells, mitogen-activated protein kinases, human gut microbiota, antiviral immunity, mucosal immunity

## Abstract

Intestinal epithelial cells (IECs) are constantly exposed to commensal flora and pathogen challenges. How IECs regulate their innate immune response to maintain gut homeostasis remains unclear. Interferons (IFNs) are cytokines produced during infections. While type I IFN receptors are ubiquitously expressed, type III IFN receptors are expressed only on epithelial cells. This epithelium specificity strongly suggests exclusive functions at epithelial surfaces, but the relative roles of type I and III IFNs in the establishment of an antiviral innate immune response in human IECs are not clearly defined. Here, we used mini-gut organoids to define the functions of types I and III IFNs to protect the human gut against viral infection. We show that primary non-transformed human IECs, upon viral challenge, upregulate the expression of both type I and type III IFNs at the transcriptional level but only secrete type III IFN in the supernatant. However, human IECs respond to both type I and type III IFNs by producing IFN-stimulated genes that in turn induce an antiviral state. Using genetic ablation of either type I or type III IFN receptors, we show that either IFN can independently restrict virus infection in human IECs. Importantly, we report, for the first time, differences in the mechanisms by which each IFN establishes the antiviral state. Contrary to type I IFN, the antiviral activity induced by type III IFN is strongly dependent on the mitogen-activated protein kinases signaling pathway, suggesting a pathway used by type III IFNs that non-redundantly contributes to the antiviral state. In conclusion, we demonstrate that human intestinal epithelial cells specifically regulate their innate immune response favoring type III IFN-mediated signaling, which allows for efficient protection against pathogens without producing excessive inflammation. Our results strongly suggest that type III IFN constitutes the frontline of antiviral response in the human gut. We propose that mucosal surfaces, particularly the gastrointestinal tract, have evolved to favor type III IFN-mediated response to pathogen infections as it allows for spatial segregation of signaling and moderate production of inflammatory signals which we propose are key to maintain gut homeostasis.

## Introduction

Intestinal epithelial cells (IECs), lining the surface of the intestine, assemble as a continuous monolayer of tightly juxtaposed cells. Their primary functions are to permit nutrient absorption and to balance electrolytes and water levels. They also act as a barrier separating the interior from the exterior milieu that enteric pathogens have to face to establish a productive infection. The lumen of the intestine is in constant contact with the “ever-present” microbiota and their various pro-inflammatory associated products (e.g., LPS). Surprisingly, this microbial load does not elicit constant inflammation in the intestine under physiological conditions. Several mechanisms have been reported to participate in the tolerance of the commensal flora. Evidence suggests that IECs generate an innate immune response in the gut that is specifically and uniquely tailored with a perfect responsive balance to flare up and control pathogens in the lumen of the gut without causing excessive local inflammation (1, 2).

Interferons (IFNs) are a class of cytokines that are often produced and secreted upon infection, in particular by viruses. IFNs bind to the infected and uninfected bystander cells to induce JAK/STAT-dependent signaling cascades that lead to the production of IFN-stimulated genes (ISGs). ISGs alert cells against the presence of pathogens conferring them an antiviral state. There are three classes of IFNs: type I, II, and III. While type II IFNs are mostly specific to immune cells, type I and III IFNs are expressed by both immune cells and epithelium cells making them very relevant for viral infection of epithelium surfaces. Type I IFNs are composed of IFN-α, IFN-β, IFN-ε, IFN-κ, and IFN-ω in humans. All type I IFNs bind to the interferon alpha receptor (IFNAR) complex, which is composed of a heterodimer of IFNAR1 and IFNAR2 (2–4). There are four subtypes of type III IFNs: IFN-λ1–3 (also called IL28a, IL28b, IL29) (5, 6) and IFN-λ4 (7). Type III IFNs bind to a heterodimeric receptor complex composed of the interferon-lambda receptor (IFNLR1) and the interleukin-10 receptor beta (6, 8, 9). Type I and III IFNs, as well as IFNAR1 and IFNAR2, are expressed by most cells. By contrast, the type III IFN-specific receptor IFNLR1 is expressed mainly on epithelial cells (i.e., respiratory tract, intestinal tract, and hepatocytes) (9–12). The functions of type I (β) and III (λ1–3) IFNs has been intensely studied in murine systems (13). However, to date, there are only few reports describing how and whether these two IFNs act differently in human organs.

The current view is that both type I and III IFNs are redundant by inducing very similar signaling pathways that lead to the expression of a comparable set of ISGs (14). This model is supported by work in the lower respiratory tract during influenza A virus (IAV) infection where multiple lines of evidence suggest that both type I and III IFNs participate in the protection against IAV (12, 15–18). On the contrary, studies focusing on the mouse gastrointestinal tract have shown an age-restricted dependence on IFNs. Neonatal mice have epithelium cells that respond to both type I and III IFNs (19), but adult mice are insensitive to type I IFNs. In adult animals, type III IFN controls local viral infection of the epithelial layer, while type I IFN controls systemic viral spread (20–23). Similarly, human hepatocytes become refractive to type I IFN treatment but never lose their ability to respond to type III IFNs (24). These examples of differential regulation of type I and type III IFNs signaling and the epithelium specificity of type III IFN-mediated immunity strongly suggest major functional and regulatory differences between the IFNs at mucosal surfaces.

Here, we use human mini-gut organoids and human IEC lines to study the relative roles of type I and type III IFNs in protecting the human gut against viral infection. We show that primary non-transformed human IECs respond to both type I and type III IFNs by producing ISGs. Using genetic ablation of either type I or type III IFN receptors, we show that either IFN can independently restrict virus infection in human IECs. However, contrary to type I IFN, the antiviral activity induced by type III IFN is strongly dependent on the mitogen-activated protein kinases (MAPKs) signaling pathway, suggesting a pathway used by type III IFNs that non-redundantly contributes to the antiviral state.

## Results

### Type III IFN Is Produced during Viral Infection of Human Mini-Gut Organoids

**Figure 1:**
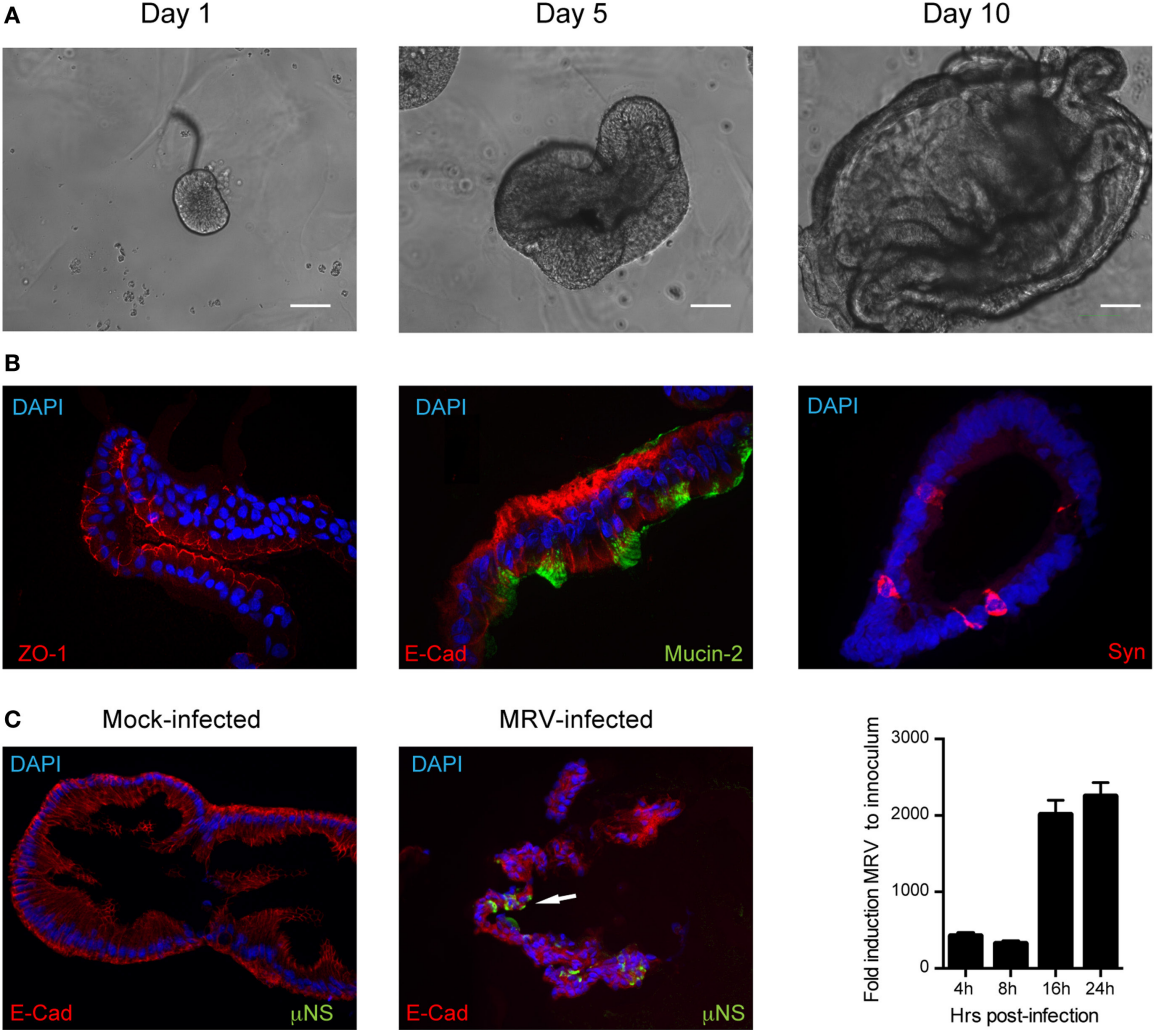
**Infection of human mini-gut organoids with mammalian reovirus (MRV)**. **(A)** Human colon organoids were prepared according to methods. Representative images of human colon organoids grown over 10 days from intestinal crypts. **(B)** Five days post-differentiation, organoids were mock or MRV infected (multiplicity of infection = 0.5). 16 hpi, organoids were fixed, cryosectioned and immunostained for adherent junctions E-cadherin (E-cad), tight junctions (ZO-1), Goblet cells (Mucin-2), and Enteroendocrine cells (synaptophysin, Syn). **(C)** MRV-infected cells were detected using an antibody against the MRV non-structural protein μNS. Representative images are shown. White arrow indicates infected cells. Organoids were infected with MRV and virus replication was monitored by qRT-PCR over a timecourse of 24 h. Data represent the mean values of three independent experiments. Error bars indicate the SD.

**Figure 2:**
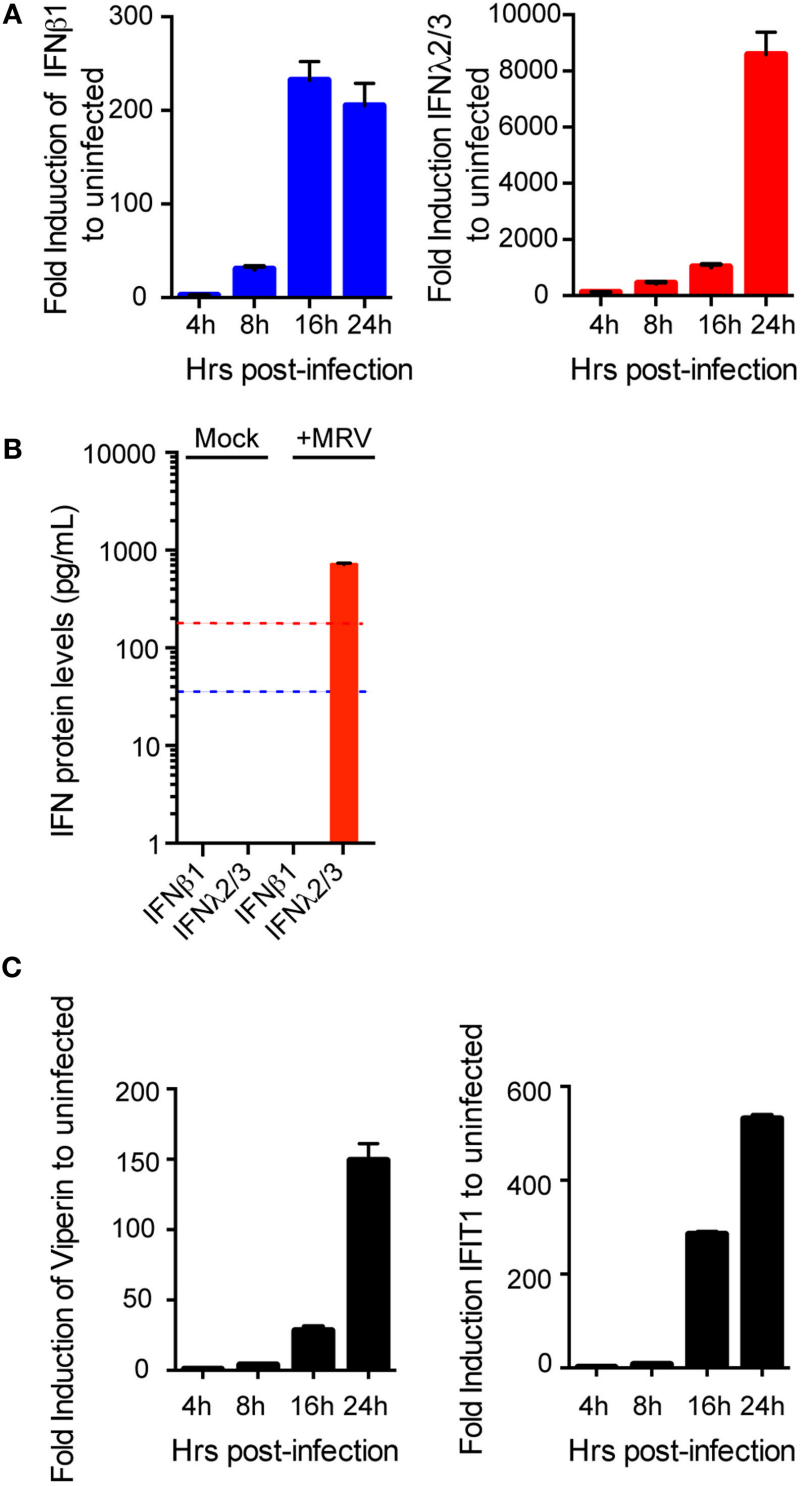
**Induction of immune response in human mini-gut organoids after mammalian reovirus (MRV) infection**. Organoids were infected with MRV (multiplicity of infection = 0.5), quantitative real-time (qRT)-PCR and ELISA were used to detect **(A)** a time course of transcriptional upregulation of both type I interferons (IFNs) (β) and type III IFN (λ2/3) IFNs **(B)** 24 hpi the production and secretion of IFN proteins in the supernatant of infected organoids and **(C)** a time course of transcriptional upregulation of the IFN-stimulated genes Viperin and IFIT1. qRT-PCR data were normalized to TBP and HPRT1 (housekeeping genes) and are expressed relative to uninfected organoids at each time point. qRT-PCR data and ELISA data represent the mean values of three independent experiments. Error bars indicate the SD. The blue and red lines in **(B)** demarcate the limit of detection of our ELISA for type I and type III IFNs, respectively.

**Figure 3:**
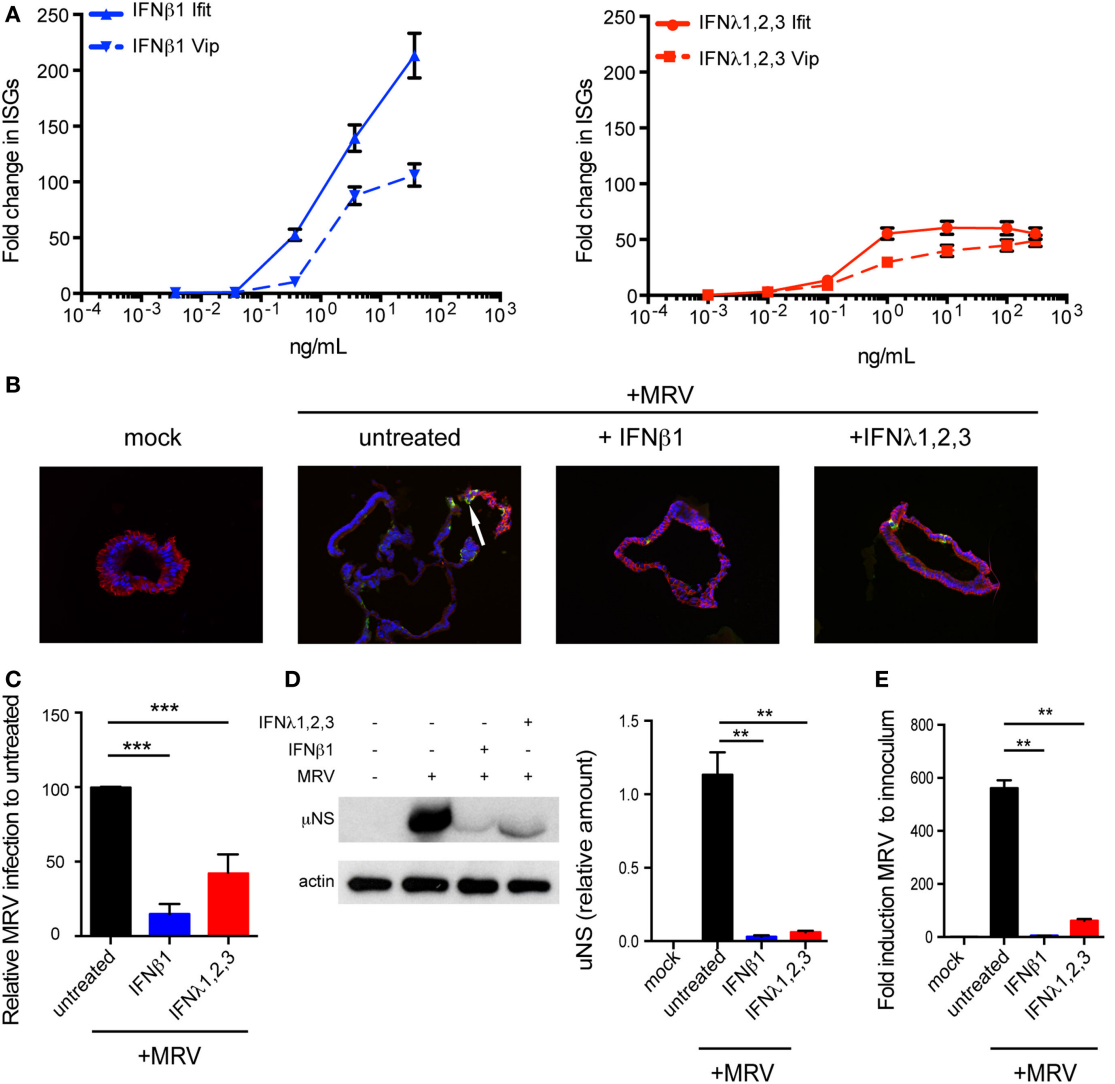
**Both type I and type III interferons (IFNs) confer human mini-gut organoids protection against viral infection**. **(A)** Colon organoids were treated with increasing concentrations of type I IFN (β) and type III IFN (λ1–3) IFN. Six hours posttreatment, organoids were harvested and the transcriptional upregulation of the IFN-stimulated genes Viperin (Vip) and Ifit1 was measured using qRT-PCR. Data were normalized to TBP and HPRT1. **(B–E)** Colon organoids were treated with type I IFN (β) (2,000 RU/mL equivalent 8 ng/mL) or type III IFN (λ1−3) (300 ng/mL) for 2.5 h prior to infection with mammalian reovirus (MRV) (multiplicity of infection = 0.5) for 16 h. **(B)** MRV-infected organoids were analyzed by μNS-specific immunofluorescence (green). The cells were stained against E-cadherin (red) and the nuclei were stained with Dapi (blue). Representative data from triplicate experiments are shown. White arrow indicates infected cells. **(C)** The fluorescence intensity of MRV μNS per organoid was measured and expressed relative to untreated organoids (set as 100). **(D)** MRV-infected organoids were analyzed for μNS production by Western blot. Actin was used as loading control. Production of μNS was quantified by densitometer. **(E)** The protective effect of type I or type III IFN was assayed by monitoring viral replication by qRT-PCR normalized to inoculum. Data represent the mean values of three independent experiments. Error bars indicate the SD. **P < 0.01, ***P < 0.001 (unpaired *t*-test).

The roles of type I and III IFNs at mucosal surfaces and in epithelial cells have been extensively studied in mice (12, 15, 16, 25, 26). Whether and how these two IFN types have antiviral activity in human epithelial cells remains much less characterized, particularly in human intestinal epithelial cells (hIECs). To investigate the functions of both IFN types in the context of primary untransformed human cells, we used human colon and intestinal mini-gut organoids. This *ex vivo* human model for the gut fully reproduces the structural architecture of the human intestinal tract and contains all major intestinal cell lineages (27, 28). Mini-gut organoids were formed by isolating intestinal crypts containing stem cells from human gut (colon or small intestine) resections from multiple donors. Single crypts were grown in Matrigel and 24 h post-isolation, opened crypts started to re-seal with the evident formation of a lumen within these organoids at 3–5 days of culture. At 7–10 days, the organoids were significantly increased in size (Figure 1A). After differentiation, human colon organoids displayed the typical organization with a clear and developed lumen, localization of E-cadherin at the basolateral side of the cells, tight junctions located at the apical side, as well as presence of mucin-secreting goblet cells (Mucin-2) and enteroendocrine cells (Syn) (Figure 1B). To address whether type I and/or type III IFNs protect the human gut against viral infection we used mammalian reovirus (MRV). MRV is a well-known virus model that induces immune response in infected cells and is sensitive to type I and III IFNs (29). In the following, we use the terms type I and type III IFNs to describe IFN β1 and IFN λ1–3, respectively. Colon organoids were infected with MRV with a multiplicity of infection (MOI) of 0.5, harvested 16 h post-infection (hpi), and viral replication was assessed by immunostaining of the reovirus non-structural protein μNS and by quantification of viral replication using quantitative real-time (qRT)-PCR. As shown in Figure 1C, MRV efficiently infects human mini-gut organoids as evidenced by the presence of MRV-infected cells (Figure 1C, left panels) and potently replicates over the course of infection (Figure 1C, right panel). Of note, MRV infection severely disrupted the structural integrity of the human mini-gut organoids (Figure 1C, also confirmed later in Figure 3B), which were mostly fragmented pieces of cellular monolayers, compared to the intact structures observed in mock-infected organoids. To characterize the innate immune response generated by organoids, we monitored the upregulation of both type I (IFN-β) and III (IFN-λ2–3) IFNs and of two representative ISGs (Viperin and IFIT1) over the course of MRV infection. We found that viral infection of organoids induces the transcriptional upregulation of type III IFN and to a lesser extent type I IFN (Figure 2A). This was congruent with the detection of only type III IFN in the supernatant of infected organoids (Figure 2B). Additionally, viral infection of organoids was associated with the upregulation of ISGs. The transcriptional upregulation of two representative ISGs (Viperin and IFIT1) over the course of MRV infection is shown in Figure 2C.

### Type I and III IFNs Protect Human Mini-Gut Organoids against Viral Infection

We found that viral infection of organoids induces the upregulation of both type I and III IFNs. To address whether both IFNs can in turn induce the expression of ISGs, mini-gut organoids were stimulated with a broad range of IFN concentrations. Results revealed that both type I and III IFNs induce the upregulation of ISGs in a dose-dependent manner (Figure 3A). Interestingly, type I IFN appears to be more potent as it induces higher expression of the Viperin and IFIT1 ISGs compared to type III IFN (Figure 3A). Similar results were found with multiple ISGs (Mx-1, ISG15, ISG54, data not shown). We also observed a continuous increase in ISG mRNA levels as the concentration of type I IFN increased, whereas ISG transcript levels quickly reached a plateau in type III IFN-treated cells.

To evaluate whether type I and/or III IFN protect primary human IECs from viral infection, organoid cultures were treated with 8 ng/mL of type I IFN (IFN-β) (equivalent 2,000 RU/mL, see Materials and Methods) or 300 ng/mL of type III IFN (IFN-λ1−3) prior to exposure to MRV. Organoids were harvested 16 hpi for analysis of viral infection/replication by immunostaining and immunoblotting against the reovirus non-structural protein μNS as well as by quantification of viral replication using qRT-PCR. Compared to mock-treated cells, pre-treatment of colon organoids with either IFN significantly reduced both the number of MRV-infected cells (Figures 3B,C, immunostaining and quantification) and the viral antigen levels within these organoids (Figure 3D). Complementarily, viral replication was severely impaired when organoids were treated with either IFNs as assayed by qRT-PCR (Figure 3E). To ensure that these findings were neither donor nor colon specific, colon organoids from different donors (colon D2–D3) and organoids derived from ileum or jejunum were similarly pre-treated with type I or III IFNs and infected with MRV. Reduced viral infection characterized by the lower expression levels of the MRV μNS protein and the decreased MRV replication was observed in colon organoids generated from different donors (Figure S1A in Supplementary Material) and in ileum (Figure S1B in Supplementary Material) and jejunum (Figure S1C in Supplementary Material) derived organoids. Similar results were found using vesicular stomatis virus (VSV), an unrelated model virus whose replication is also sensitive to both type I and III IFNs (29, 30). Pre-treatment of human organoids with either IFNs resulted in a significant inhibition of VSV replication as measured by the significant decrease of bioluminescence when using VSV-expressing luciferase (VSV-luc) as a reporter of viral replication (Figure S2 in Supplementary Material). All together, these results demonstrate the antiviral protective role of both type I and III IFNs in colon, ileum, and jejunum derived hIECs.

### Human IEC Lines Express Type I and III IFNs upon MRV Infection

**Figure 4:**
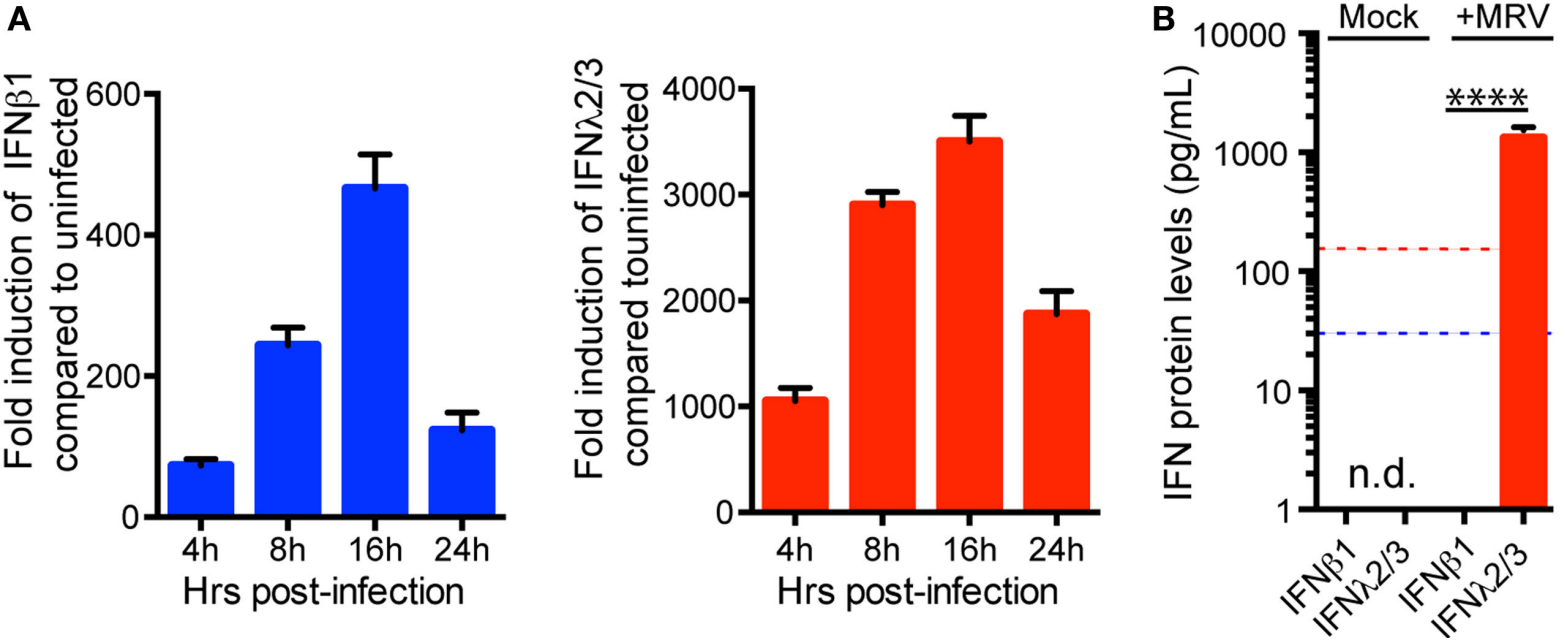
**Expression pattern of interferon (IFN) mRNA and protein in human intestinal epithelial cells upon viral infection (A)**. Relative quantification of type I IFN (β) and type III IFN (λ2/3) transcripts during the course of mammalian reovirus (MRV) (multiplicity of infection = 1) infection of T84 cells. Data are normalized to TBP and HPRT1 and are expressed relative to uninfected cells at each time point. **(B)** Quantification of type (IFNβ) and type III (IFN λ2/3) protein levels by ELISA in supernatants of uninfected or MRV-infected T84 cells. The blue and red dashed lines demarcate the limit of detection of our ELISA for type I and type III IFNs, respectively. n.d., not detectable. Data represent the mean values of three independent experiments. Error bars indicate the SD. ****P < 0.0001 (unpaired *t*-test).

Human mini-gut organoids are very difficult to modify genetically. Therefore, in order to better characterize the functions and the mechanisms by which type I and III IFNs confer hIECs an antiviral state, we used the human colon carcinoma-derived cell line T84. T84 cells were infected with MRV and harvested at different time points post-infection to evaluate the transcriptional upregulation of both type I and III IFNs. Viral infection of T84 cells induces the upregulation of type I and type III IFNs (Figure 4A). Similar to human mini-gut organoids (Figure 2), viral infection induces a higher transcriptional upregulation of type III IFNs compared to type I IFN (Figure 4A; Figure S3A in Supplementary Material). To address whether both IFNs were made at the protein level and secreted by infected T84 cells, we measure the amount of both IFNs in the supernatant of infected T84 cells using ELISA. As observed for viral infection of mini-gut organoids (Figure 2B), only type III IFN was found in the supernatant (Figure 4B). However, type I IFN can be detected in the supernatant if added exogenously to inhibit viral infection (data not shown).

To address whether T84 IECs respond to either type I and III IFNs, we treated T84 IECs with type I or III IFN and measured the expression levels of ISGs at different time points post-IFN treatment. Like human mini-gut organoids (Figure 3A), we found that type I IFN induces higher expression of the ISGs Viperin and IFIT1 compared to type III IFN (Figure S3B in Supplementary Material). Transcriptome analysis of T84 cells treated with either type I or III IFN revealed that type I IFN consistently induced higher transcript levels across all induced ISGs (Figure S3C in Supplementary Material).

**Figure 5:**
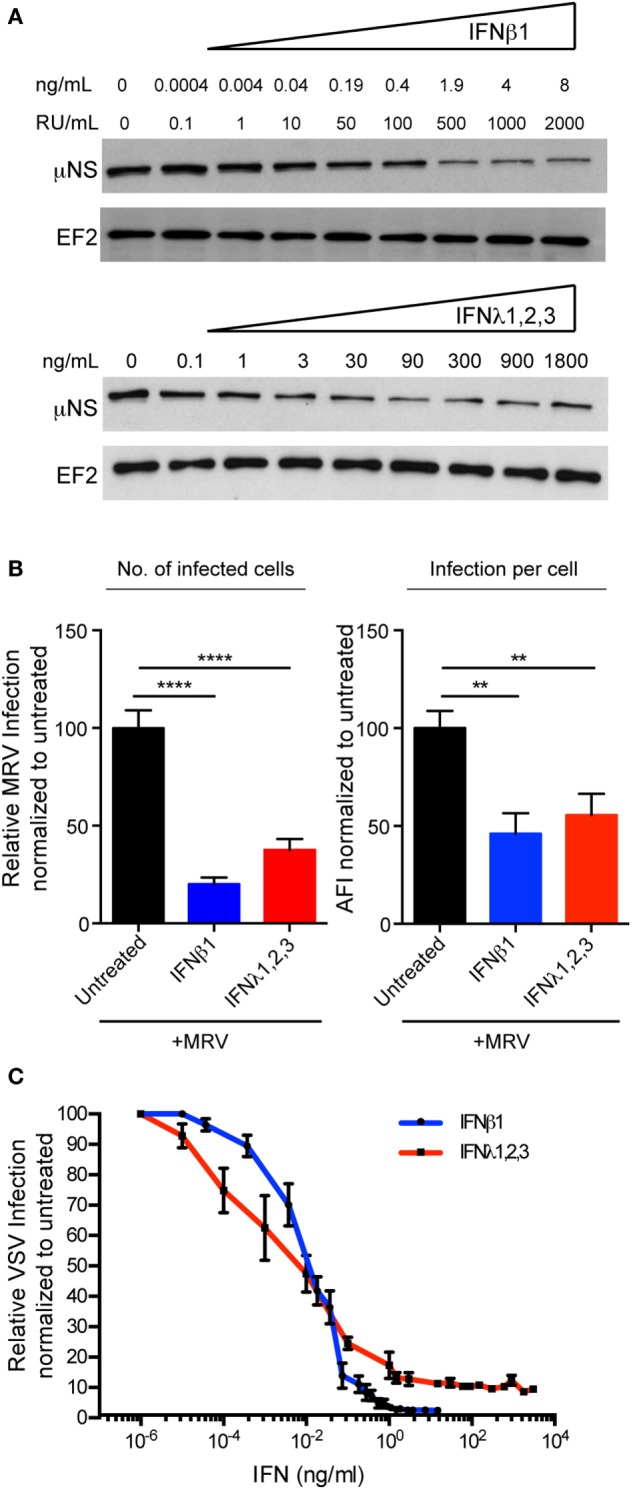
**Both type I and type III interferons (IFNs) mediate antiviral protection in human T84 cells**. **(A)** T84 cells were pre-treated for 2.5 h with the indicated concentrations of type I IFN (β) and type III IFN (λ1–3) IFNs and then subsequently infected with mammalian reovirus (MRV) [multiplicity of infection (MOI) = 1]. Sixteen hours post-infection, the protective effect of type I or III IFN was assayed by immunoblotting for the viral non-structural protein μNS. EF-2 is used as a loading control. A representative immunoblot out of three independent experiments is shown. **(B)** T84 cells were treated with type I IFN (β) (2,000 RU/mL equivalent 8 ng/mL) or type III IFN (λ1−3) (300 ng/mL) for 2.5 h prior to infection with MRV for 16 h. MRV-infected cells were analyzed by μNS-specific immunofluorescence. (Left panel) The number of infected cells was quantified and is expressed relative to untreated cells (set to 100). (Right panel) MRV uNS staining intensity was measured to obtain the average fluorescent intensity per cell and is expressed relative to untreated cells (set to 100). Data represent the mean values of three independent experiments. **(C)** T84 cells were pre-treated with the indicated concentrations of type I or III IFNs for 2 h prior to infection with vesicular stomatis virus (VSV) expressing Firefly luciferase VSV expressing luciferase (MOI = 1). Viral replication was assayed by measuring the luciferase activity. For each sample luciferase activity was measured in triplicates and is expressed as the percentage of the activity present in VSV-infected cells without IFN treatment (set to 100). The mean value obtained from three independent experiments is plotted. Error bars indicate the SD. **P < 0.005, ****P < 0.0001 (unpaired *t*-test).

To determine the antiviral potency of type I and III IFNs in T84 cells, we pre-treated T84 cells with increasing concentrations of each IFN prior infection with MRV. *De novo* production of viral proteins was monitored by blotting for the MRV non-structural protein μNS. Figure 5A shows that both type I and III IFNs inhibit viral infection in a dose-dependent manner. In addition, cells were fixed 16 hpi and the fraction of μNS-expressing cells was determined by immunofluorescence, which demonstrates that either type I or III IFN decreased both the number of MRV-infected cells and the level of viral antigen per cell (Figure 5B). To confirm that this observation was not virus-specific, T84 cells were treated with increasing concentrations of either type I or III IFNs and subsequently infected with VSV-luc as a reporter of viral replication. Measurement of viral infection by bioluminescence showed that similar to MRV, either type I or III IFNs are capable of inhibiting VSV infection in hIECs in a dose-dependent manner (Figure 5C). All together these results show that either type I or III IFNs confer T84 cells lines an antiviral state and that T84 cells phenocopy the antiviral response generated by primary hIECs in the context of mini-gut organoid.

### Type I and III IFN Signaling Pathways Independently Mediate an Antiviral State in Human IECs

**Figure 6:**
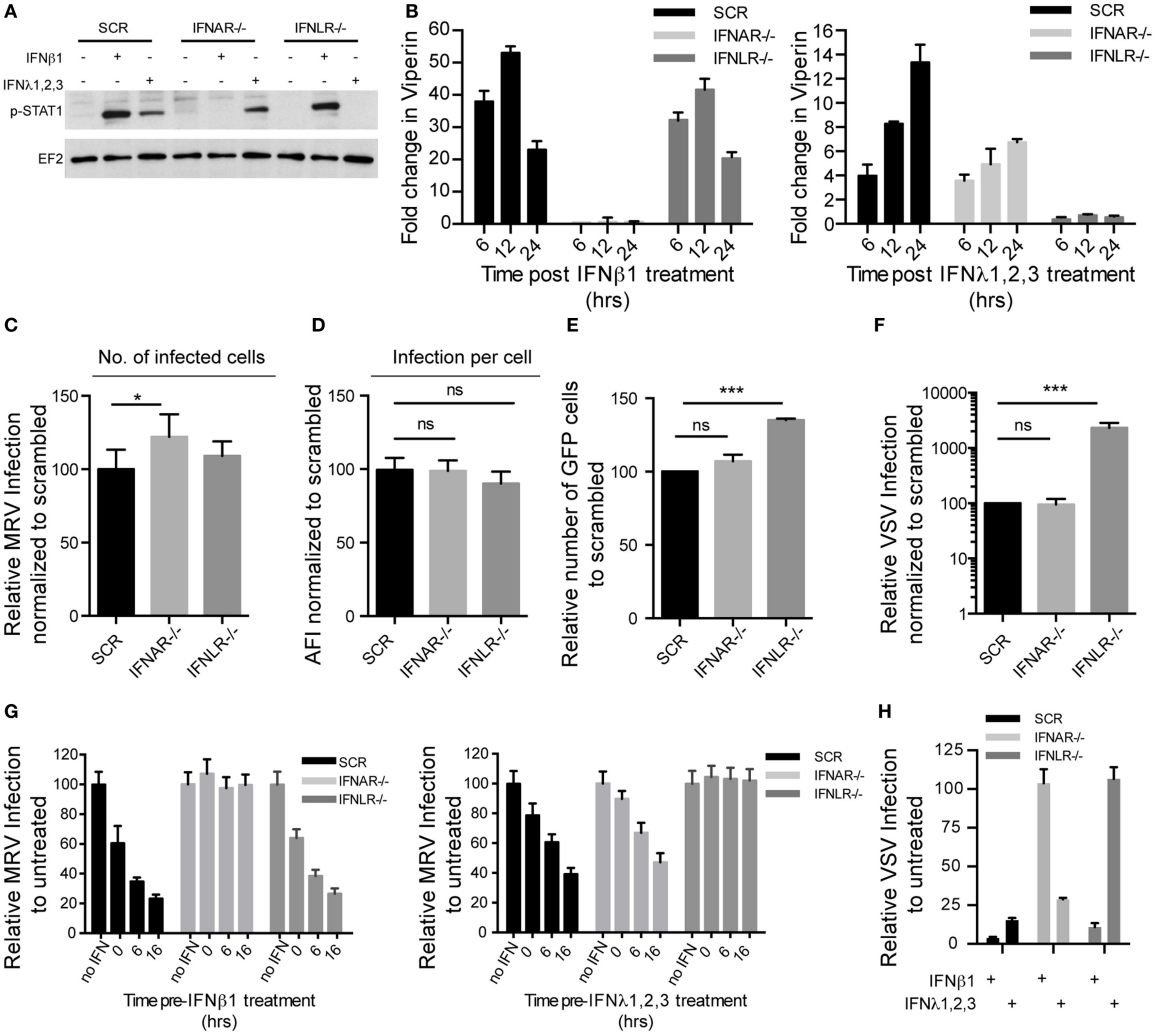
**Type I and type III interferons (IFNs) independently confer intestinal epithelial cells antiviral protection**. T84 IFNAR1 and IFNLR1 knockout cell lines were generated using the CRISPR/Cas9 system. **(A)** T84 cell lines were treated with type I IFN (β) (2,000 RU/mL equivalent 8 ng/mL) or type III IFN (λ1−3) (300 ng/mL) for 1 h and IFN signaling was measured by immunoblotting for pSTAT1 Y701. EF-2 is used as a loading control. A representative immunoblot out of three independent experiments is shown. **(B)** Same as **(A)**, except that induction of IFN-stimulated genes was monitored by relative qRT-PCR quantification of Viperin at indicated times post-IFN treatment. Data were normalized to TBP and HPRT1 and are expressed relative to untreated cells of each time point. **(C,D)** T84 cell lines were infected with mammalian reovirus (MRV) for 16 h (multiplicity of infection (MOI) = 1) and MRV-infected cells were analyzed by μNS-specific immunofluorescence. **(C)** The number of infected cells is expressed relative to scramble control cells (set to 100). **(D)** MRV μNS staining intensity was measured to obtain the average fluorescence intensity per cell and expressed relative to scramble control cells (set to 100). **(E)** T84 cell lines were infected with vesicular stomatis virus (VSV)-GFP (MOI = 1) for 8 h and the number of VSV-infected cells were analyzed by FACS. The percentage of infected cells is expressed relative to scramble control cells (set to 100). **(F)** Same as **(E)**, except that T84 cell lines were infected with VSV expressing luciferase (VSV-luc) (MOI = 1) and viral replication was assayed by measuring the luciferase activity. For each cell line luciferase activity was measured in triplicates and is expressed relative to scramble control cells (set to 100). **(G)** Same as **(C)**, except that T84 cell lines were treated with type I IFN (β) (2,000 RU/mL equivalent 8 ng/mL) or type III IFN (λ1−3) (300 ng/mL) at indicated time points prior to infection with MRV. **(H)** Same as **(F)**, except that T84 cell lines were treated with type I IFN (β) (2,000 RU/mL equivalent 8 ng/mL) or type III IFN (λ1−3) (300 ng/mL) for 2 h prior to infection with VSV-luc. Data **(B–H)** represent the mean values of three independent experiments. Error bars indicate the SD. *P < 0.05, ***P < 0.001, ns, not significant (unpaired *t*-test).

To test whether types I and III IFNs act in combination or separately in establishing the antiviral state of IECs, we generated T84 cell lines deficient for either the IFN alpha (IFNAR) or the IFN lambda (IFNLR) receptor using CRISPR/Cas9 technology. Inactivation of the IFN receptors was confirmed by sequencing of the knockout (KO) cell lines, which revealed nucleotide deletions and changes of open reading frame in IFNAR−/− and IFNLR−/− genes (data not shown). As shown in Figures 6A,B, IFNAR−/− cells were no longer able to phosphorylate pSTAT1 and induce ISGs after type I IFN treatment, but remained fully responsive to type III IFN, indicating a selective disruption of the type I IFN signaling pathway. Conversely, IFNLR−/− cells were insensitive to type III IFN but responded to type I IFN. These results were consistent across multiple IFNAR−/− and IFNLR−/− cell clones (Figures S4A,B in Supplementary Material).

To evaluate whether deletion of IFNAR or IFNLR renders human IECs more susceptible to viral infection, cells lacking functional receptors for type I or III IFN were infected by either MRV or VSV and compared to wild-type or scrambled guide RNA-exposed cells. Immunofluorescence analysis revealed that loss of IFNAR slightly increased the number of MRV-infected cells compared to control cells (Figure 6C), but did not affect the average fluorescent intensity of MRV antigen per infected cell (Figure 6D). Interestingly, IFNLR−/− cells appeared to be more susceptible to VSV infection. The number of VSV-infected cells and the amount of viral antigens in each cells were significantly increased in IFNLR−/− cells compared to control cells (Figures 6E,F). To confirm the protective role of type I and III IFN against viral infection in human IECs, IFNAR−/− and IFNLR−/− cells were pre-treated with either type I or III IFNs and subsequently infected with MRV or VSV. Type I or III IFN could efficiently inhibit infection by both MRV and VSV in control cells (Figures 6G,H). As expected, the protective effect of type I IFN against MRV (Figure 6G, left panel) and VSV (Figure 6H) was no longer observed in IFNAR1−/− cells, but was preserved in IFNLR1−/− cells. Conversely, disruption of IFNLR1−/− specifically abolished the protective effect of type III IFN, but not of type I IFN. Similar results were obtained with several KO clones (Figure S4C in Supplementary Material). All together these data demonstrate that in human T84 cells, either type I or III IFNs are capable of independently mediating antiviral protection.

### MAP Kinases Are Required for Type III but Not Type I IFN Antiviral Activity in hIECs

**Figure 7:**
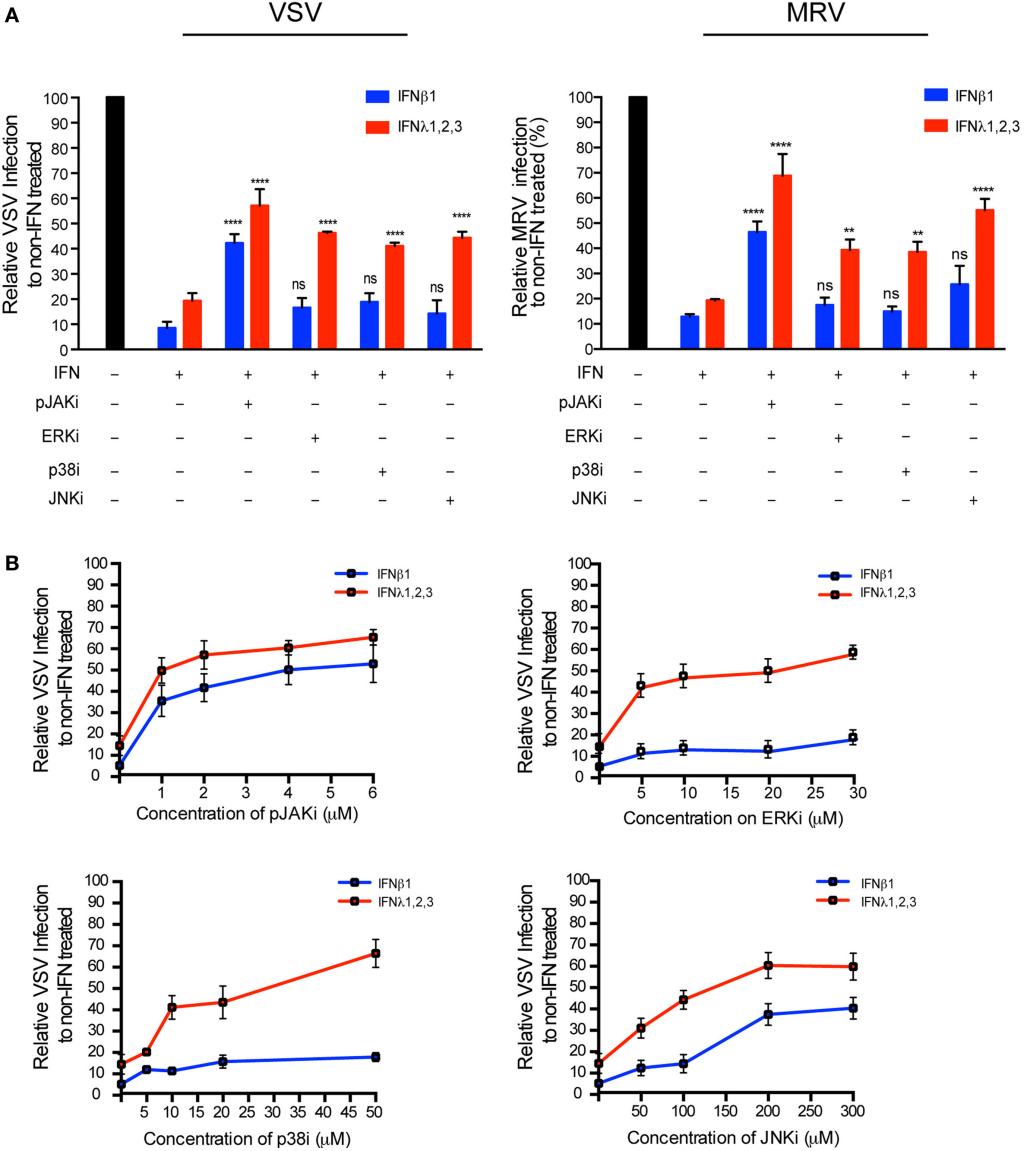
**Type III interferons (IFNs) require mitogen-activated protein kinases for their antiviral response**. **(A)** T84 cells were mock incubated (black bar) or pre-incubated for 30 min with 2 μM Pyridone 6 (pan-JAK inhibitor), 10 μM U0126 (ERK inhibitor), 10 μM SB202190 (p38 inhibitor), or 100 μM SP600125 (JNK inhibitor). Then, T84 cells were mock treated (black bar) or treated with type I IFN (β) (2,000 RU/mL equivalent 8 ng/mL) or type III IFN (λ1−3) (300 ng/mL) in the presence the inhibitor. Two hours post-IFN treatment cells were infected with a multiplicity of infection of 1 with VSV expressing luciferase (left panel) or mammalian reovirus (right panel). Viral replication was assayed by measuring the luciferase activity or by relative quantification of viral genome using qRT-PCR. Data were normalized to non-IFN-treated sample for each inhibitor (set to 100). **(B)** Same as **(A)**, except T84 cells were pre-incubated with increasing concentrations of JAK or MAP kinase inhibitors prior to treatment with IFNs. The mean value obtained from three independent experiments, is plotted. Error bars indicate the SD. ****P < 0.0001, **P < 0.005, ns, not significant (unpaired *t*-test).

Type I and III IFN signaling and antiviral activity are dependent on the JAK/STAT pathway, and inhibition of STAT1 phosphorylation blocks the production of ISGs and inhibits IFN-mediated antiviral protection (31–33). Several MAPKs have also been reported to be activated (34) and contribute to ISG upregulation in type I or III IFN-stimulated cells (35, 36), but the role of the MAPK pathways in the antiviral functions of type III IFN remains unclear. We found that both type I or III IFN treatment induced the phosphorylation of STAT1, STAT2, and STAT3 with similar kinetics in T84 cells (data not shown). Type I or III IFN treatment did not induce STAT5A, STAT5B, or STAT6 phosphorylation (data not shown). We next addressed whether type I and III IFNs activate the MAPKs. We found that both IFNs induce the phosphorylation of the MAPKs, p38, ERK, and JNK to the same extent and with similar kinetics (Figure S5A in Supplementary Material). To determine the role of the STAT and MAPK pathways in the antiviral activity of IFNs, we used specific pharmacological inhibitors in combination with IFN treatment. The specificity of these inhibitors and their toxicity were tested in T84 cells by Western blot analysis (Figure S6 in Supplementary Material) and cell viability assay (Figure S5B in Supplementary Material). Inhibiting the JAK/STAT pathway with a pan-JAK inhibitor almost fully blocked phosphorylation of STAT1 (Figure S6 in Supplementary Material) and strongly impaired the antiviral activity of either type I or III IFNs on both VSV and MRV (Figure 7A). Interestingly, inhibition of the MAPKs with specific inhibitors, had no effect on the phosphorylation kinetics of STAT1 (Figure S6 in Supplementary Material) but strongly affected the antiviral protection conferred by type III IFN only (Figure 7A). This specific inhibition is seen across a range of concentrations (Figure 7B). Of note, a partial inhibition of the antiviral activity of type I IFN was observed at high concentration of JNK inhibitor (Figure 7B) but at this concentration cell viability was severely affected (Figure S5B in Supplementary Material). This type III IFN restricted dependence on MAPKs was independent of IFN concentration (Figure S5C in Supplementary Material), validating that the non-dependence of type I IFN for MAPKs was not the result of differences in the IFN concentration used to stimulate the cells. Altogether, these results demonstrate the fundamental role of STAT-dependent signaling in conferring both type I and type III IFNs antiviral activity, and in addition demonstrate a unique role for MAPKs toward inducing the antiviral state induced by type III IFN but not type I IFN.

**Figure 8:**
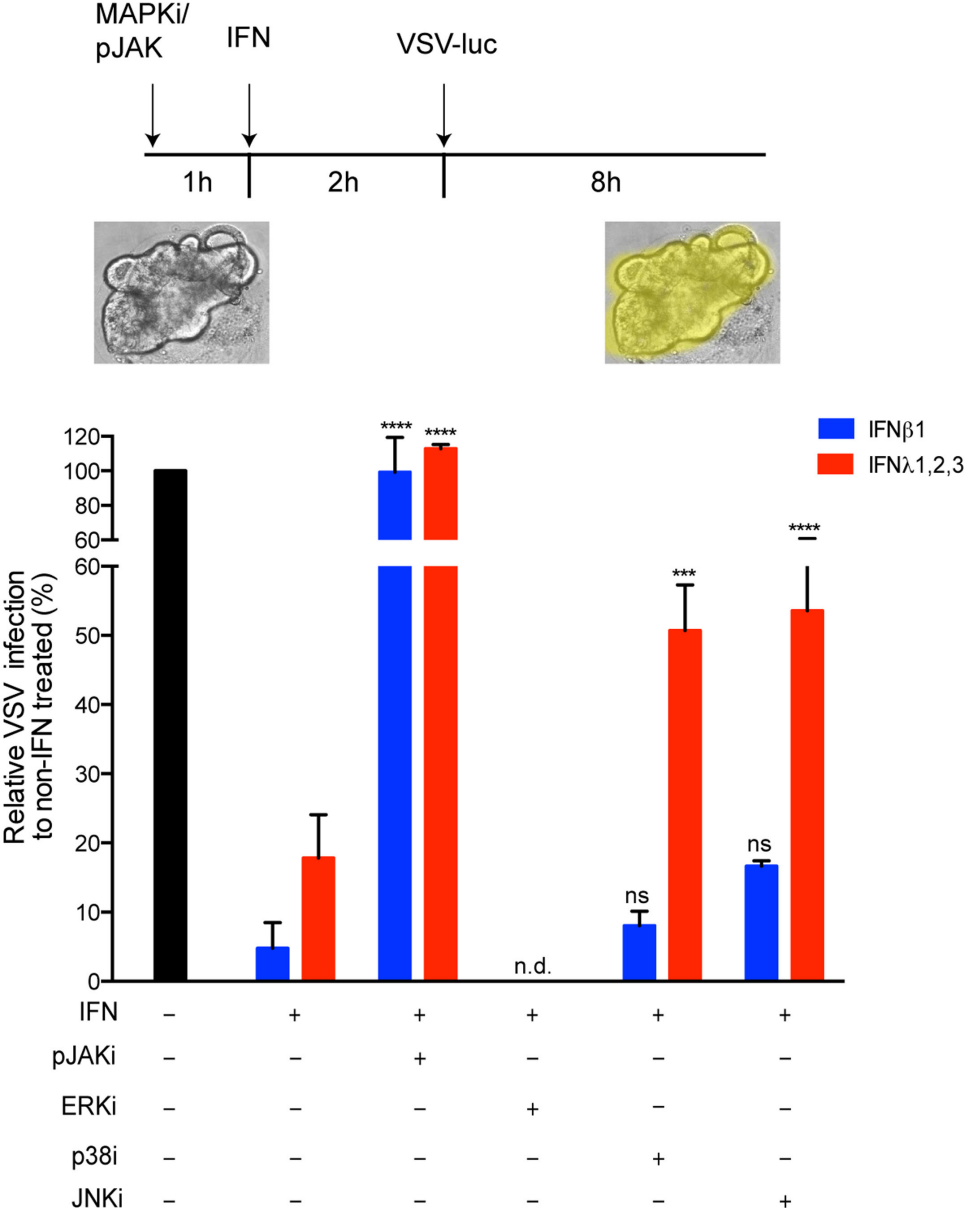
**The antiviral activity of type III interferons (IFNs) strongly dependent on mitogen-activated protein kinases in the contact of primary human intestinal epithelial cells**. Human colon organoids were mock incubated (black bar) or pre-incubated with 2 μM Pyridone 6 (pan-JAK inhibitor), 10 μM U0126 (ERK inhibitor), 10 μM M SB202190 (p38 inhibitor) and 100 μM SP600125 (JNK inhibitor). One hour posttreatment, organoids were mock treated (black bar) or co-treated with type I IFN (β) (2,000 RU/mL equivalent 8 ng/mL) or type III IFN (λ1−3) (300 ng/mL) for 2 h. Organoids were then infected with VSV expressing luciferase (multiplicity of infection = 1). Eight hpi, viral replication was assayed by measuring the luciferase activity. Data are normalized to non-IFN-treated sample for each inhibitor (set to 100). The mean value obtained from three independent experiments is plotted. Error bars indicate the SD. ****P < 0.0001, ***P < 0.001, ns, not significant (unpaired *t*-test), n.d. (not determined).

Although it has been shown in multiple cell lines that IFNs can induce the activation of MAPKs (34–36), the importance of these kinases in the IFN-mediated antiviral state has never been reported to our knowledge. This suggests that dependency on MAPKs might be cell type specific. To ensure that the antiviral activity of type III IFN in primary non-transformed hIECs depends on MAPKs, we used our mini-gut organoid culture system. Colon organoids were treated with pharmacological inhibitors of the JAK/STAT or MAPKs signaling pathways. Following pre-treatment with type I or type III IFNs, organoids were infected with VSV. Eight hpi, organoids were harvested and the impact of the pharmacological inhibitors on the antiviral activities of both IFNs was measured. As expected, inhibition of the JAK/STAT signaling pathway fully restores VSV infection to a level similar to infected organoids in the absence of IFNs (Figure 8). This confirms that the JAK/STAT signaling pathways is key for both type I and type III IFN activity in primary hIECs. Interestingly and similar to T84 cells, inhibition of either p38 or JNK MAPKs partially impairs only the antiviral activity of type III IFNs in human mini-gut organoids (Figure 8). No significant effect of MAPK inhibition on type I IFN-mediated antiviral activity was observed. The effect of inhibiting ERK-dependent signaling on the antiviral activity of both IFNs was not determined (n.d.) since treatment of mini-gut organoids with ERK inhibitor induced disruption and death of the organoid culture (Figure 8 and data not shown). Altogether, these results confirm that MAPK signaling pathways participate in the establishment of the antiviral state mediated by type III IFN in primary non-transformed hIECs.

## Discussion

In recent years, there has been a large interest in uncovering the specific roles of type III IFNs in epithelial cells including lung epithelium, gastrointestinal tract epithelium and in hepatocytes. In this work, by exploiting human mini-gut organoids, we performed a functional characterization of both type I and III IFNs in a human primary intestinal cell context. We found that, upon viral infection, human IECs strongly upregulate both type I and III IFNs at the transcriptional level. Although only type III IFN was found to be secreted by IECs, we demonstrated that either type I or III IFNs induce the production of ISGs and that this production is associated with the establishment of an antiviral state that efficiently protects IECs from viral infection. Importantly, we revealed that type III IFN-mediated signaling allows for efficient protection against viral infection while limiting ISG production. We propose that this represents a mechanism to limit inflammation in the gut while remaining responsive to pathogens. Additionally, genetic ablation of IFN signaling using CRISPR/Cas9-mediated KO of IFN receptors further demonstrated that type I and III IFNs independently mediate an antiviral activity. Comparative analyses revealed that both IFNs induce the same set of ISGs and that both antiviral states depend on the JAK/STAT signaling pathway. Importantly, we discovered that the type III IFN-mediated, but not the type I IFN-mediated antiviral activity depends on MAPK signaling pathways. This work establishes that both type I and III IFNs provide potent antiviral protection in the human gut, and identifies, for the first time, fundamental differences in the mechanism by which these two IFN types establish the antiviral state in primary hIECs.

Since the implementation of organoid cultures, these systems have gained substantial and increasing interest in the fields of cellular biology and medicine (27, 28). More recently, these organoids have been also used to study and describe infectious diseases (37–41). In the present study, we have exploited organoids not only to describe the response of hIECs upon pathogen challenges but also to perform a functional characterization of both type I and III IFNs in the context of primary non-transformed hIECs.

In contrast to data in murine IECs (20), our results demonstrate that hIECs can mount an antiviral state in response to either type I or III IFN treatment. Similar observations have been made in lung epithelium, where both type I and type III IFNs participate in the protection against IAV (12, 15–18). Our functional characterizations performed in both mini-gut organoids and colon carcinoma-derived cell lines revealed that although these cells transcriptionally upregulate both type I and III IFNs, they secrete very little to no type I IFN. A favored type III IFN response over type I IFN has been observed in other epithelial cells stimulated with various viruses and pathogen-associated molecular patterns (16, 30). It was shown that although both IFNs can protect airway epithelial cells against viral infection, type III IFNs were preferentially made in response to influenza infection (15). Similarly, it was shown that upon stimulation of IECs with the double strand RNA structural analog poly-inosinic:cytidylic acid (poly I:C), only type III IFN was secreted by the cells although both type I and III IFNs were upregulated at the transcriptional level (42). Additionally, it has been reported that human hepatocytes can become refractive to type I IFN, while maintaining their responsiveness to type III IFN (24). Consequently, favoring type III IFN signaling appears to be a common strategy developed at epithelial surfaces (airway, hepatocytes, intestinal tract) to mount an antiviral response.

It remains unclear whether translation or secretion of type I IFN is restricted in hIECs. To date, very little is known about the mechanisms that lead to type I and III IFN secretion. It has been shown that signaling downstream of mitochondrial-associated MAVS (mitochondrial antiviral-signaling protein) induces the secretion of type I IFN that can be inhibited by brefeldin A. On the contrary, the antiviral activity generated following activation of peroxisomal-associated MAVS was insensitive to brefeldin A (29). It was later demonstrated that this briefeldinA-insensitive antiviral state was mediated by type III IFN, which was secreted following activation of peroxisomal MAVS (30). These observations strongly suggest that type I and III IFNs are secreted from cells by two distinct mechanisms.

Although type III IFN stimulation of hIECs results in significantly less induction of ISGs compared to type I IFN (Figure 3; Figure S3 in Supplementary Material), we found that type III IFN was only slightly less potent in protecting the cell against viral infection (Figures 3 and 5). This lower induction of ISGs is not cell type specific as recent publications addressing the role of these IFNs in hepatocytes also reported that type III IFN induces less ISGs compared to type I IFN (43–45). However, in these studies, the antiviral potency of both IFNs was not addressed side-by-side. As such, type III IFN could be considered a milder IFN favored at epithelial surfaces (at least intestinal epithelium) due to its ability to confer an antiviral state without inducing excessive amounts of ISGs, which might result in the induction of local pro-inflammatory signals. The molecular mechanisms by which type III IFN signaling modulates ISG expression remain unknown. Desensitization is a possible mechanism through which IFN signaling is reduced following stimulation. Different negative regulators of IFN signaling might be used to regulate or turn off ISG expression. Signal transduction strongly depends on the amount of receptor and the affinity of the ligand for its receptor. It is known that type I IFN (IFNβ) has a very strong affinity for its receptor. Differences in the affinity of type I and III IFNs for their respective receptor or differences in the amount of type I and III IFN receptors at the surface of hIECs might be partially responsible for the observed differences in the magnitude of ISG expression.

Functional characterization of type III IFN and comparison to type I IFN suggests that both cytokines are functionally redundant by inducing the same set of ISGs (8, 35, 46). However, the restriction of type III IFN receptor to epithelium cells suggests that type III IFN might have unique functions or provide specific advantages at epithelial surfaces. Several studies have tried to characterize functional differences both in human (hepatocytes) (43, 44) and in murine model systems (lung and intestinal tract) (16, 19, 20, 26). To date, the main difference between both IFNs has been explained by the spatial restriction of type III IFN receptor at epithelial surfaces. In this work, we demonstrate that type III IFN induces less ISGs compared to type I IFN. Most importantly, we unravel, for the first time, fundamental differences in the mechanisms by which both IFN mount the antiviral state in hIECs. We demonstrate that the antiviral activity of type III IFN partially depends on MAPKs, which is not the case for type I IFN. Interestingly, inhibition of MAPKs did not influence the expression of both IFIT1 and Viperin ISGs (data not shown). As such, it remains an important task for future work to dissect how signaling downstream of MAPKs participates in the antiviral activity of type III IFN only. As both IFNs have been reported to activate MAPKs (34–36), it will be interesting to address whether the dependency of type III IFN for MAPKs is epithelium cell specific or intestinal epithelium cell specific.

It is not known whether hIECS can protect themselves against viral infection by secreting and responding to their own IFNs. It was proposed that, during rotavirus infection, IFNs are produced by immune cells and not by epithelial cells (42). Indeed, during rotavirus infection of hIECs, multiple strategies are developed by the virus to inhibit innate immune response particularly the inhibition of both type I and III IFNs production (47). Additionally, blocking IFN signaling in hIECs does not lead to an increased rotavirus replication (42). Our data clearly show, for the first time, that when primary hIECs are infected with viruses that do not block IFN synthesis, hIECs produce and secrete at least type III IFN (maybe some type I IFN but under the detection limit of our ELISA assay) in order to protect themselves. Complementarily, KO of IFNLR renders hIECs more susceptible to viral infection (Figure 6).

Considering our results that only type III IFN is secreted by hIECs, it is tempting to propose that IECs have evolved to favor type III IFN over type I IFN, as it allows for similar protection against pathogens while limiting production of ISGs. From the perspective of an epithelium, which is always exposed to the extracellular environment and commensal challenges, this might represent a “smart strategy” to regulate the immune response in order to achieve the balance between responsiveness to pathogens versus tolerance of commensals. Restricting signaling to type III IFNs allows for response compartmentalization because type III IFN signaling is limited mostly to epithelial cells (11, 12, 35), thereby limiting systematic inflammation. From our findings in epithelium cells of the gastrointestinal tract, we can speculate that the first response to pathogen threats will be generated by hIECs. This response will be characterized by type III IFN-mediated signaling, therefore limiting ISGs and pro-inflammatory cytokine production. This first wave response of type III IFN produced by IECs, alone might be enough to clear enteric virus infection (22, 48). A second wave response might be generated through recruitment of immune cells at the site of epithelium infection, which in turn will produce various cytokines including type I IFN. This IFN will then mediate a strong induction of ISGs and pro-inflammatory signals to powerfully combat pathogen at the infected mucosa and also will provide systemic protection. This uniquely tailored response would be fundamental for the maintenance of human gut homeostasis.

## Materials and Methods

### Cells, Viruses, and Viral Infection

T84 human colon carcinoma cells (ATCC CCL-248) were maintained in a 50:50 mixture of Dulbecco’s modified Eagle’s medium and F12 (GibCo) supplemented with 10% fetal bovine serum and 1% penicillin/streptomycin (Gibco). Reovirus MRV strains Type 3 clone 9 derived from stocks originally obtained from Bernard N. Fields were grown and purified by standard protocols (49). VSV-luc was a kind gift from Sean Whelan (Harvard Medical School) and was produced as described in Ref. (50). An MOI of 1 was used to infect T84 cells and organoids. Titers were determined as described in Ref. (51). For T84 cell MRV infections, MRV were purified on CsCl-gradient and stocks were titred by fluorescence foci forming assay (express in FFU) in T84 cells. Titers were calculated by determining the 50% tissue culture infective dose and expressed in FFU/mL. T84 cells were infected as described in Ref. (49). The MOI was determined as the ratio of infected cells (determined by fluorescence foci forming assay)/total number of cells. An MOI of 1 was used in T84 based experiments resulting in about 50–60% of infected as determined by fluorescence assay. For mini-gut organoids MRV infection, Organoids were removed from Matrigel by adding cold-PBS for 5 min, liquefied Matrigel and organoids were separated by centrifugation (400 g 5 min), the total number of cells per organoid samples was measured using an haematocytometer. Organoids were resuspended in culture medium containing or not MRV. When using an MOI of 1 (as determined in T84 cells) to infect mini-gut organoids, very few infected cells were detected per organoid. This discrepancy between T84 and organoid infectivity might be due to the 3-dimensional nature of the organoids and to residual Matrigel that might absorb and neutralize MRV. As such MRV stocks were titred directly in organoids by serial dilution infection and subsequent immunostaining. The MOI was calculated by the ratio of the number of infected/total number of cells/organoid. An MOI of 0.5 was used to infect organoids.

### Human Organoid Cultures

Human colon tissue was received from colon resection (52–54) from the University Hospital Heidelberg under the approved study protocol S-024/2003 and human ileum and jejunum were purchased from Baylor University and transferred by signed MTA. Stem cells containing crypts were isolated following 2 mM EDTA dissociation of tissue sample for 1 h at 4°C. Crypts were spun and washed in ice-cold PBS. Fractions enriched in crypts were resuspended in Matrigel and maintained in basal culture media (53) Advanced DMEM/F12, supplemented with 1% penicillin/streptomycin, 10 mM HEPES, 50% v/v Wnt3A conditioned media, 1× B-27 (Life technology), 1× N-2 (Life technology), 2 mM GlutaMax (Gibco), 50 ng/mL EGF (Invitrogen), 1 µg/mL Spondin (Peprotech), 100 ng/mL Noggin (Peprotech), 10 nM Gastrin (Sigma), 1 mM *N*-acetyl-cysteine (Sigma), 10 mM nicotinamide (Sigma), and 500 nM A-83-01 (Tocris). Differentiation media is the same as above except without Wnt3A, nicotinamide and 50% reduced levels of R-Spondin and Noggin. Organoids were stained after cryo-sectioning of embedded organoids in Tissuetek.

### Antibodies/Reagents

Rabbit polyclonal antibody against reovirus μNS was used at a 1/1,000 dilution for immunostaining and Western blots (49). Commercially available primary antibodies were goat polyclonal antibody recognizing EF-2 (Santa Cruz Biotechnology # sc-13004), rabbit polyclonal anti-Mucin-2 (Santa Graz Biotechnology# sc-15334), mouse monoclonal antibodies recognizing phospho-STAT1 or STAT1 (BD Transductions #612233 or #610115, respectively), ZO-1 (Invitrogen #339100) or E-cadherin (BD Transductions #610181). Rabbit polyclonal anti-phospho p38 (#4511), anti-p38 (#8690), anti-phospho-SAPK/JNK (#4668), anti-SAPK/JNK(#9258), anti-phospho ERK1/2 (#4370), and anti-ERK1/2 (#4695) antibodies were obtained from Cell Signaling. Secondary antibodies were conjugated with AF568 (Molecular Probes) or horseradish peroxidase (HRP) (Sigma-Aldrich) and directed against the animal source. Anti-mouse (GE Healthcare # NA934V), anti-rabbit (GE Healthcare #NA931V) and anti-goat (Jackson Immunoresearch # 705-035-147) antibodies, each coupled with HRP, were used as secondary antibodies for Western blot at a 1:5,000 dilution. Human recombinant IFN-beta1a (IFNβ) was obtained from Biomol (#86421). Recombinant human IFNλ 1 (IL-29) (#300-02L), IFNλ 2 (IL28A) (#300-2K), and IFNλ 3 (IL-28B) (#300-2K) were purchased from Peprotech. The pharmacological inhibitors used were 2 µM Pyridone 6 (Calbiochem #420099-500), 10 μM SB202190 (Tocris Bioscience #1264) for p38, 100 μM SP600125 (Tocris Bioscience #1496) for JNK, and 10 μM U0126 (Cell signaling #9903) for MEK-1/2.

### RNA Isolation, cDNA, and qPCR

RNA was harvested from cells using the NucleoSpin RNA extraction kit (Machery-Nagel) and following the manufacturer’s instructions. cDNA was made using iSCRIPT reverse transcriptase (Bio-Rad) from 250 ng of total RNA as per the manufacturer’s instructions. qRT-PCR was performed using SsoAdvanced SYBR green (Bio-Rad) as per the manufacturer’s instructions. TBP and HPRT1 were used as normalizing genes. Type I IFN was analyzed using primers specific for human IFNβ, and type III IFN was analyzed using primers specific for human IFNλ 2/3. The expression levels (fold of induction) of the investigated genes were calculated as ΔΔCq, normalizing to untreated or mock samples and to normalizing genes.

### Western Blot

At time of harvest, media was removed, cells were rinsed once with 1× PBS and lysed with 1× RIPA (150 mM sodium chloride, 1.0% Triton X-100, 0.5% sodium deoxycholate, 0.1% sodium dodecyl sulfate (SDS), 50 mM Tris, pH 8.0 with phosphatase, and protease inhibitors (Sigma-Aldrich)) for 5 min at room temperature (RT). Lysates were collected and equal protein amounts were separated by SDS-PAGE and blotted onto a PVDF membrane by wet-blotting (Bio-Rad). Membranes were blocked with 5% milk or 5% BSA in TBS containing 0.1% Tween 20 (TBS-T) for 1 h at RT. Primary antibodies were diluted in blocking buffer and incubated overnight at 4°C. Membranes were washed 3× in TBS-T for 5 min at RT. Secondary antibodies were diluted in blocking buffer and incubated at RT for 1 h with rocking. Membranes were washed 3× in TBS-T for 5 min at RT. HRP detection reagent (GE Healthcare) was mixed 1:1 and added to the membrane, which was then incubated at RT for 5 min. Membranes were exposed to film and developed.

### Indirect Immunofluorescence Assay

T84 cells were seeded in a 24-well plate. Cells were fixed in 2% paraformaldehyde for 20 min at RT, washed with PBS and permeabilized using 0.5% Triton X-100 for 15 min. After blocking with 3% BSA in PBS for 1 h at RT, cells were incubated with primary antibodies in 3% BSA for 1 h at RT. After washing with PBS, cells were stained with secondary antibodies in 3% BSA for 45 min at RT. To stain mini-gut organoids, 10 µm cryosections were fixed in 80% ethanol for 10 min at RT, followed by 2 min incubation in ice-cold acetone. After blocking in 5% goat serum in PBS containing 1% Triton for 1 h at RT, sections were incubated with primary antibodies in blocking solution for 2 h at RT or overnight at 4°C. After washing in PBS, sections were stained with secondary antibodies in 1% BSA in PBS containing 0.5% Triton for 2 h at RT. Nuclear DNA was stained with ProLong Gold DAPI (Molecular Probes). Slides were imaged by epifluorescence using a Nikon Eclipse Ti-S (Nikon) microscope or by confocal tile scans on a Zeiss LSM 780 (Zeiss) microscope. Image processing was performed using the Fiji software. For infection experiments, the percentage of infected cells was determined by counting at least 600–1,000 cells detected in 10 fields of view for each condition.

### VSV Luciferase Assay

T84 cells were seeded in a white bottom 96-well plate. Cells were pre-treated prior to infection as indicated with increasing concentrations of type I or type III IFNs. VSV-luc (MOI = 1) was added to the wells and the infection was allowed to proceed for 8 h. At the end of the infection, media was removed, cells were washed 1× with PBS and lysed with Cell Lysis Buffer (Promega) at RT for 5 min. 1:1 dilution of Steady Glo (Promega) and PBS were added to the cells and incubated at RT for 7 min. Luminescence was read using an Omega Luminometer.

### Microarray

Total RNA was purified as described above from T84 cells treated with 2,000 RU/mL of type I IFN (β) or 100 ng/mL of each type III IFN (λ1−3) for 6 hr. Microarray data were processed using the software package R. Differentially expressed probe sets were determined by comparing the triplicate stimulated samples with the three unstimulated samples. Significance was defined by a minimum absolute of twofold change in expression and a q-value (false discovery rate) <0.05.

### ELISA

IFNβ and IFNL2/3 contained in the supernatant of cells were quantified using the human IFN-beta ELISA kit and DIY IFNLR 2/3 ELISA kit both from PBL-Interferon Source, per manufacturer’s instructions.

### Human KO Cell Lines

Knockout of IFNAR1 and IFNLR1 in T84 cells were achieved by using the CRISPR/Cas9 system. Three different single-guide RNAs (sgRNAs) per gene were used targeting the coding region of IFNAR1 and IFNLR1 and inserted into the lentiviral vector lentiCRISPR v2 (Addgene #52961) also encoding the Cas9 nuclease. The following sgRNAs were used: IFNAR1 (#1) 5′ GCGGCTGCGGACAACACCCA 3′, (#2) 5′ GACCCTAGTGCTCGTCGCCG 3′, (#3) 5′CTAGGGTCGTCGCGCCCAGG3′, IFNLR1(#1) 5′ACTGGATCTGAAGTATGAGG3′, (#2) 5′CCTGGTGCTCACCCAGACGG3′ (#3) 5′ TGAGGTGGCATTCTGGAAGG 3′. Lentiviruses were produced and T84 cells were transduced two times using 1:2 diluted stocks of lentiviral particles encoding sgRNA #1, 2 or 3. All shown data were obtained by using a cell clone treated with the sgRNA #2 for IFNAR1 and IFNLR1, but analogous results were obtained with cell clones generated with the other sgRNAs. To establish IFNAR1 and IFNLR1 KO cells, clonal selection was performed via single-cell dilution in a 96-well plate. KOs were confirmed by functional tests.

## Ethics Statement

This study was carried out in accordance with the recommendations of “University hospital Heidelberg” with written informed consent from all subjects. All subjects gave written informed consent in accordance with the Declaration of Helsinki. The protocol was approved by the “Ethic commission of University hospital Heidelberg” under the approved study protocol S-024/2003.

## Author Contributions

MS, KP, and SB designed the experiments. KP, MS, SM, and DA performed most experiments. LR and RR performed IFN-specific qRT-PCR. SK and JS-B assisted with organoid preparation. ES and DG designed CRISPR/cas experiments. MS, RR, DG, and SB wrote the manuscript.

## Conflict of Interest Statement

The authors declare that the research was conducted in the absence of any commercial or financial relationships that could be construed as a potential conflict of interest.

## Supplementary Material

Figure S1 **Type I and III interferons (IFNs) confer protection against mammalian reovirus (MRV) infection to all sections of the gut**. Organoids from multiple colon donors and multiple intestinal sections were treated with type I IFN (β) (2,000 RU/mL equivalent 8 ng/mL) or type III IFN (λ1−3) (300 ng/mL) for 2.5 h prior to infection with MRV (multiplicity of infection = 0.5) for 16 h. **(A)** MRV-infected colon organoids (donor 2 and 3) were analyzed by μNS-specific immunofluorescence (green). The cells were stained against E-cadherin (red) and the nuclei were stained with Dapi (blue). Representative image of triplicate experiments are shown. The fluorescence intensity of MRV μNS per organoid was measured and expressed relative to untreated organoids (set to 100). **(B,C)** MRV-infected organoids were analyzed for μNS production by Western blot. Actin was used as loading control. Production of μNS was quantified by densitometer. The protective effect of type I IFN (β) and III IFN (λ1−3) was assayed by monitoring the relative viral genome copies by quantitative real-time PCR normalized to inoculum. **(B)** Ileum. **(C)** Jejunum. Data represent the mean values of three independent experiments. Error bars indicate the SD. ***P* < 0.01, ****P* < 0.001 (unpaired *t*-test).

Figure S2 **Type I and III interferons (IFNs) confer protection against vesicular stomatis virus (VSV) infection in mini-gut organoids**. Colon organoids were treated with type I IFN (β) (2,000 RU/mL equivalent 8 ng/mL) or type III IFN (λ1−3) (300 ng/mL) for 2 h prior to infection with VSV-expressing luciferase (multiplicity of infection = 1). Eight hpi, VSV replication was assayed by measuring the luciferase activity. The mean values obtained from three independent experiments are plotted. Error bars indicate the SD.

Figure S3 **T84 cells respond to both type I and III interferons (IFNs) by upregulating IFN-stimulated genes (ISGs)**. **(A)** T84 cells were infected with mammalian reovirus (multiplicity of infection = 1) and 16 hpi, cells were harvested and the copy number of the expression of 13 human type I and three type III IFNs by quantitative real-time (qRT)-PCR (55) were determined by qRT-PCR analysis. The geometric means of the peak responses in mock and infected intestinal epithelial cells are shown in a log10 scale as copy numbers per μg RNA. **(B)** T84 cells were stimulated with indicated concentrations of type I (β) or III IFN (λ1−3) for different times and the transcript levels of the ISGs Viperin (Vip) and IFIT1 were analyzed by qRT-PCR. Data are normalized to TBP and HPRT1 and are expressed relative to untreated cells at each time point. A representative experiment out of three independent experiments is shown. Mean values and SD are shown. **(C)** T84 cells were treated with type I IFN (β) (2,000 RU/mL equivalent 8 ng/mL) or type III IFN (λ1−3) (300 ng/mL) for 6 h and identification of the IFN-induced ISGs was performed by transcript profiling using an Illumina microarray.

Figure S4 **Characterization of different T84 IFNAR and IFNLR knockout (KO) cell clones generated using the CRISPR/Cas system**. **(A)** IFNAR and IFNLR KO clones were treated with type I interferons (IFN) (β) (2,000 RU/mL equivalent 8 ng/mL) or type III IFN (λ1−3) (300 ng/mL) for 1 h and IFN signaling was measured by immunoblotting for pSTAT1 Y701. EF-2 is used as a loading control. A representative immunoblot out of three independent experiments is shown. **(B)** Same as **(A)**, except that IFN signaling was evaluated by monitoring induction of IFN-stimulated genes by relative quantification of Viperin at indicated times post-IFN treatment using qRT-PCR. Data are normalized to TBP and HPRT1 and are expressed relative to untreated control cells of each time point. A representative experiment, out of three independent experiments is shown. **(C)** T84 cell lines were treated with type I IFN (β) (2,000 RU/mL equivalent 8 ng/mL) or type III IFN (λ1−3) (300 ng/mL) for 2 h prior to infection with VSV-expressing luciferase (multiplicity of infection = 1) and viral replication was assayed by measuring the luciferase activity. Results are expressed relative to mock-IFN-treated control cells generated with a scrambled control gRNA (set to 100). The mean value obtained from two independent experiments is plotted. Error bars indicate the SD.

Figure S5 **Role of MAP kinase pathway in type I and III interferon (IFN) antiviral activity**. **(A)** T84 cells were treated with type I IFN (β) (2,000 RU/mL equivalent 8 ng/mL) or type III IFN (λ1−3) (300 ng/mL) for the indicated time points. The levels of phosphorylation of MAPkinases p38, ERK, and JNK were assessed by Western blot analysis. p38, ERK, JNK, and EF-2 were used as loading control. The phosphorylation of the MAP kinases was quantified and expressed relative to untreated cells (right panel). Data represent the mean values of three independent experiments. **(B)** T84 cells were treated with increasing concentrations of JAK and MAP kinase inhibitors. 24 h posttreatment of inhibitors the cell viability was assessed by MTT assay in triplicates. **(C)** T84 cells were pre-incubated for 30 min with 2 μM Pyridone 6 (pan-JAK inhibitor), 10 μM U0126 (ERK inhibitor), 10 μM SB202190 (p38 inhibitor) and 100 μM SP600125 (JNK inhibitor). Then the indicative concentrations of type I or III IFN were added in parallel to the inhibitor. Two hours post-IFN treatment cells were infected with VSV expressing luciferase (multiplicity of infection = 1) and viral replication was assayed by measuring the luciferase activity. Data were normalized to in no IFN-treated samples for each inhibitor. The mean value obtained from three independent experiments is shown. Error bars indicate the SD.

Figure S6 **Specific inhibition of MAP kinases phosphorylation**. T84 intestinal epithelial cells were pre-treated with JAK and MAPK inhibitors for 30 min prior to interferon treatment. Cells were harvested at different times posttreatment and the extent of JAK or MAPK inhibition was addressed by Western blot analysis. The specificity of each inhibitor was controlled by monitoring the phosphorylation status of JAK and all MAPKs. **(A)** 2 μM Pyridone 6 (pan-JAK inhibitor). **(B)** 10 μM U0126 (ERK inhibitor). **(C)** 10 μM SB202190 (p38 inhibitor).
