# Supplementary figures and images for: Type I and Type III Interferons Display Different Dependency on Mitogen-Activated Protein Kinases to Mount an Antiviral State in the Human Gut
Source: Front Immunol. 2017 Apr 21;8:459. doi: 10.3389/fimmu.2017.00459 (PMC5399069; doi:10.3389/fimmu.2017.00459)

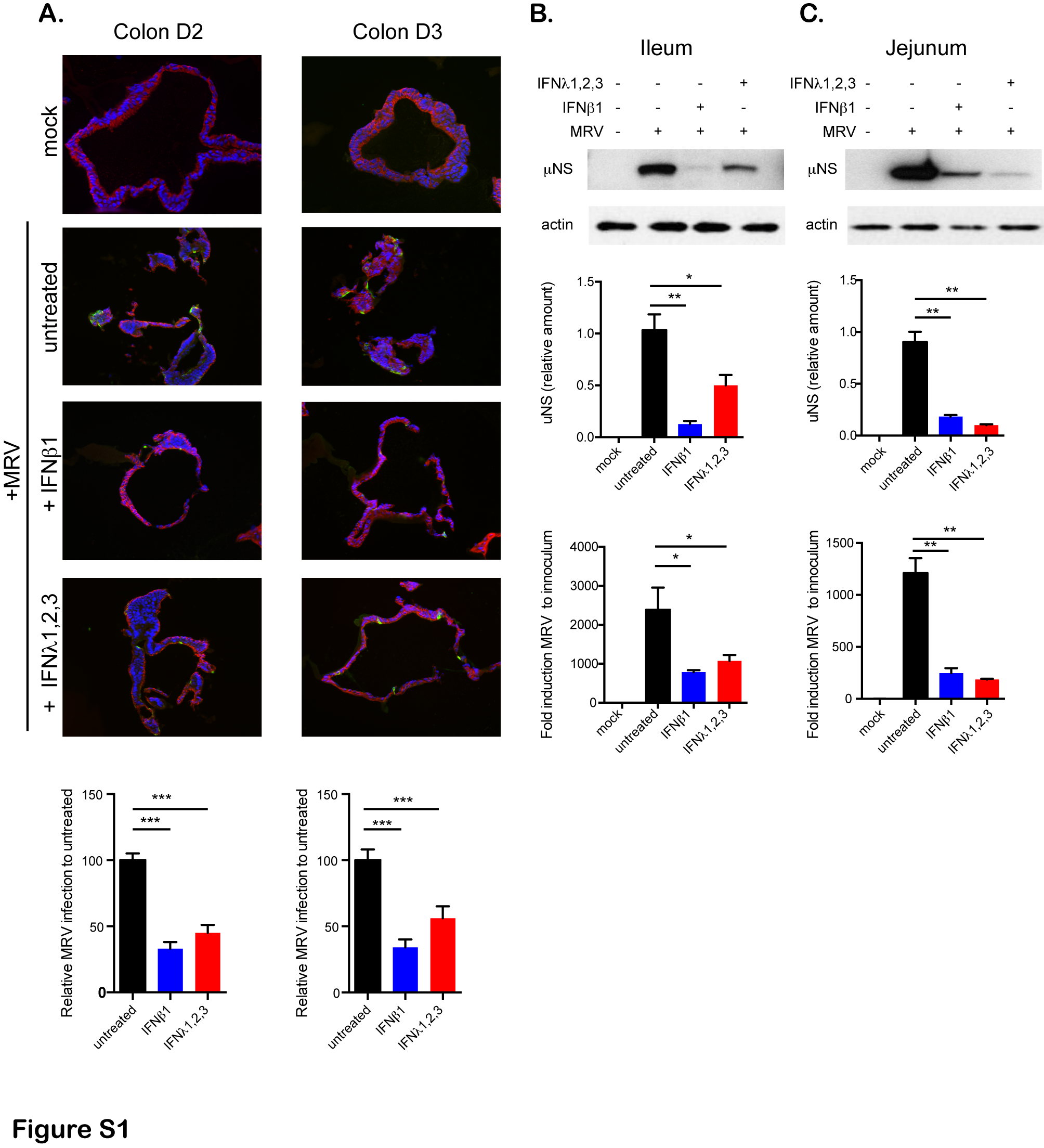

Supplement: Figure S1 — Type I and III interferons (IFNs) confer protection against mammalian reovirus (MRV) infection to all sections of the gut. Organoids from multiple colon donors and multiple intestinal sections were treated with type I IFN (β) (2,000 RU/mL equivalent 8 ng/mL) or type III IFN (λ1−3) (300 ng/mL) for 2.5 h prior to infection with MRV (multiplicity of infection = 0.5) for 16 h. (A) MRV-infected colon organoids (donor 2 and 3) were analyzed by μNS-specific immunofluorescence (green). The cells were stained against E-cadherin (red) and the nuclei were stained with Dapi (blue). Representative image of triplicate experiments are shown. The fluorescence intensity of MRV μNS per organoid was measured and expressed relative to untreated organoids (set to 100). (B,C) MRV-infected organoids were analyzed for μNS production by Western blot. Actin was used as loading control. Production of μNS was quantified by densitometer. The protective effect of type I IFN (β) and III IFN (λ1−3) was assayed by monitoring the relative viral genome copies by quantitative real-time PCR normalized to inoculum. (B) Ileum. (C) Jejunum. Data represent the mean values of three independent experiments. Error bars indicate the SD. **P < 0.01, ***P < 0.001 (unpaired t-test). [file Image_1.TIF]

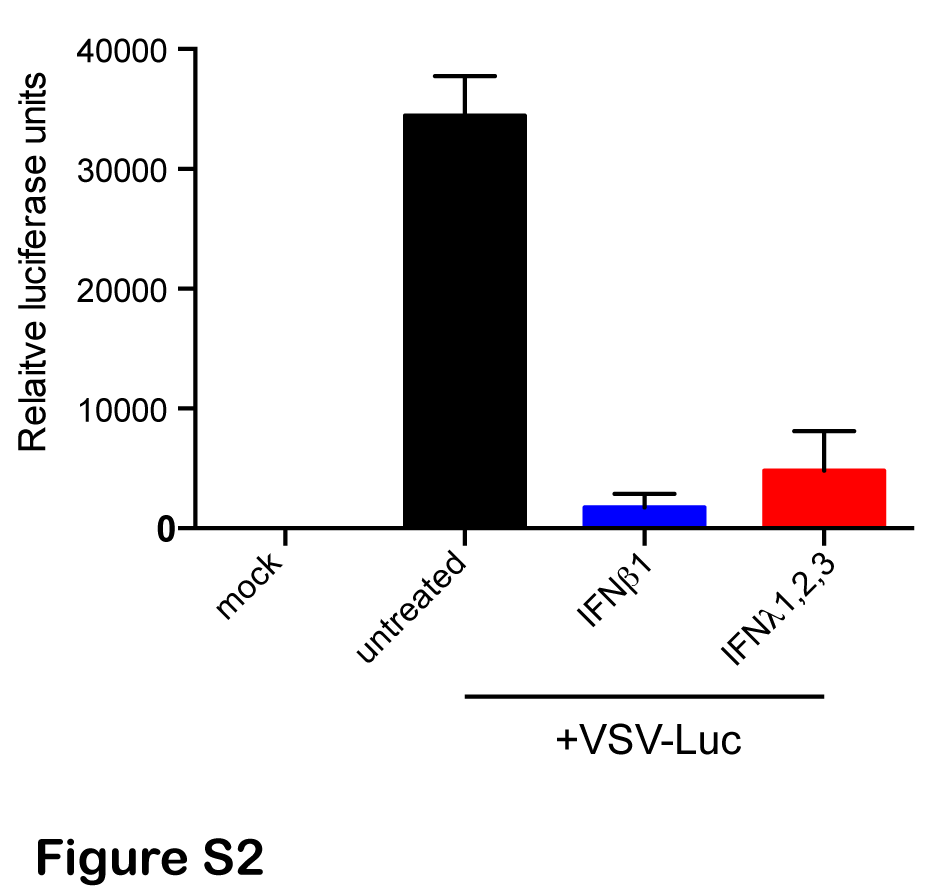

Supplement: Figure S2 — Type I and III interferons (IFNs) confer protection against vesicular stomatis virus (VSV) infection in mini-gut organoids. Colon organoids were treated with type I IFN (β) (2,000 RU/mL equivalent 8 ng/mL) or type III IFN (λ1−3) (300 ng/mL) for 2 h prior to infection with VSV-expressing luciferase (multiplicity of infection = 1). Eight hpi, VSV replication was assayed by measuring the luciferase activity. The mean values obtained from three independent experiments are plotted. Error bars indicate the SD. [file Image_2.TIF]

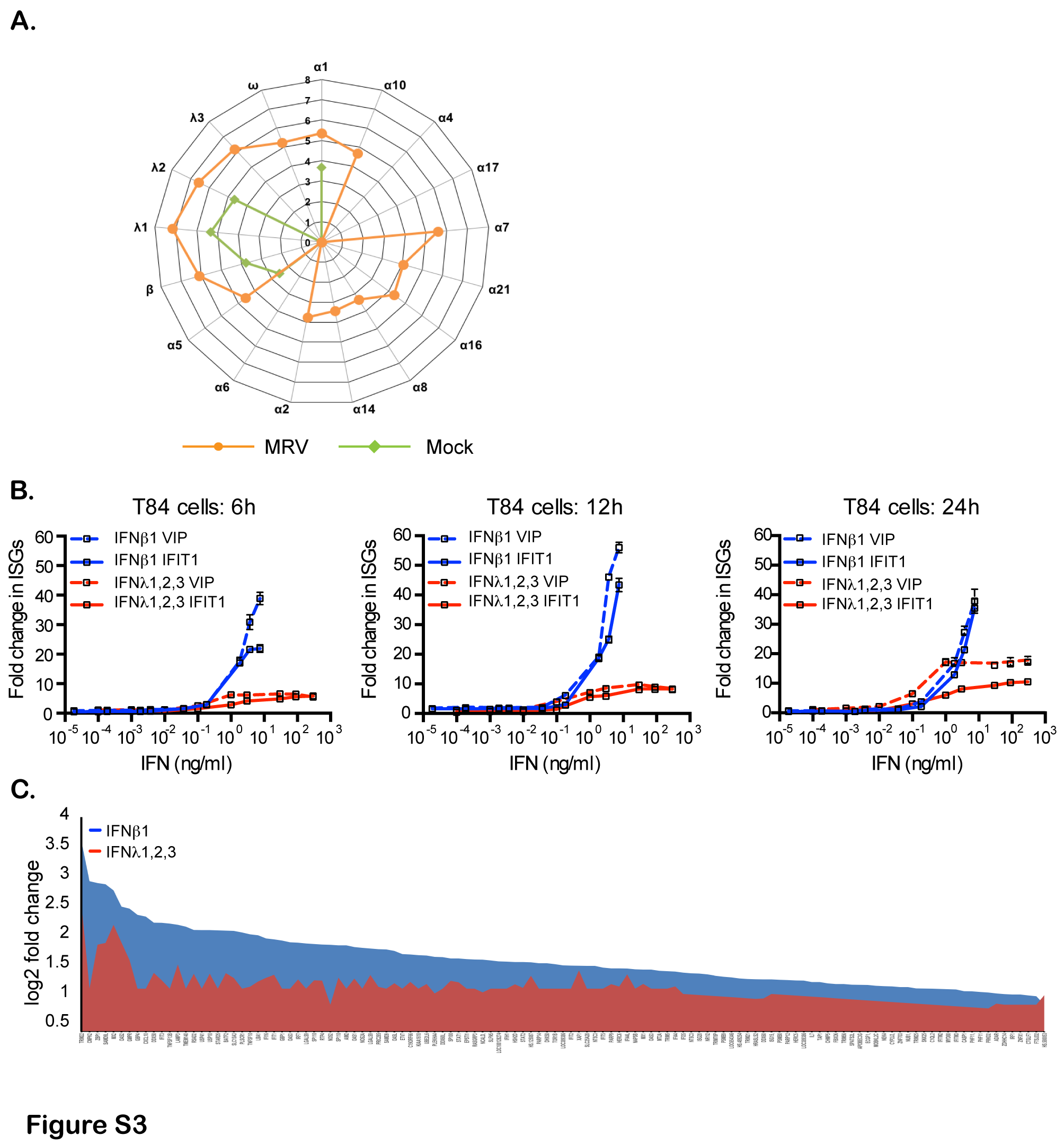

Supplement: Figure S3 — T84 cells respond to both type I and III interferons (IFNs) by upregulating IFN-stimulated genes (ISGs). (A) T84 cells were infected with mammalian reovirus (multiplicity of infection = 1) and 16 hpi, cells were harvested and the copy number of the expression of 13 human type I and three type III IFNs by quantitative real-time (qRT)-PCR (55) were determined by qRT-PCR analysis. The geometric means of the peak responses in mock and infected intestinal epithelial cells are shown in a log10 scale as copy numbers per μg RNA. (B) T84 cells were stimulated with indicated concentrations of type I (β) or III IFN (λ1−3) for different times and the transcript levels of the ISGs Viperin (Vip) and IFIT1 were analyzed by qRT-PCR. Data are normalized to TBP and HPRT1 and are expressed relative to untreated cells at each time point. A representative experiment out of three independent experiments is shown. Mean values and SD are shown. (C) T84 cells were treated with type I IFN (β) (2,000 RU/mL equivalent 8 ng/mL) or type III IFN (λ1−3) (300 ng/mL) for 6 h and identification of the IFN-induced ISGs was performed by transcript profiling using an Illumina microarray. [file Image_3.TIF]

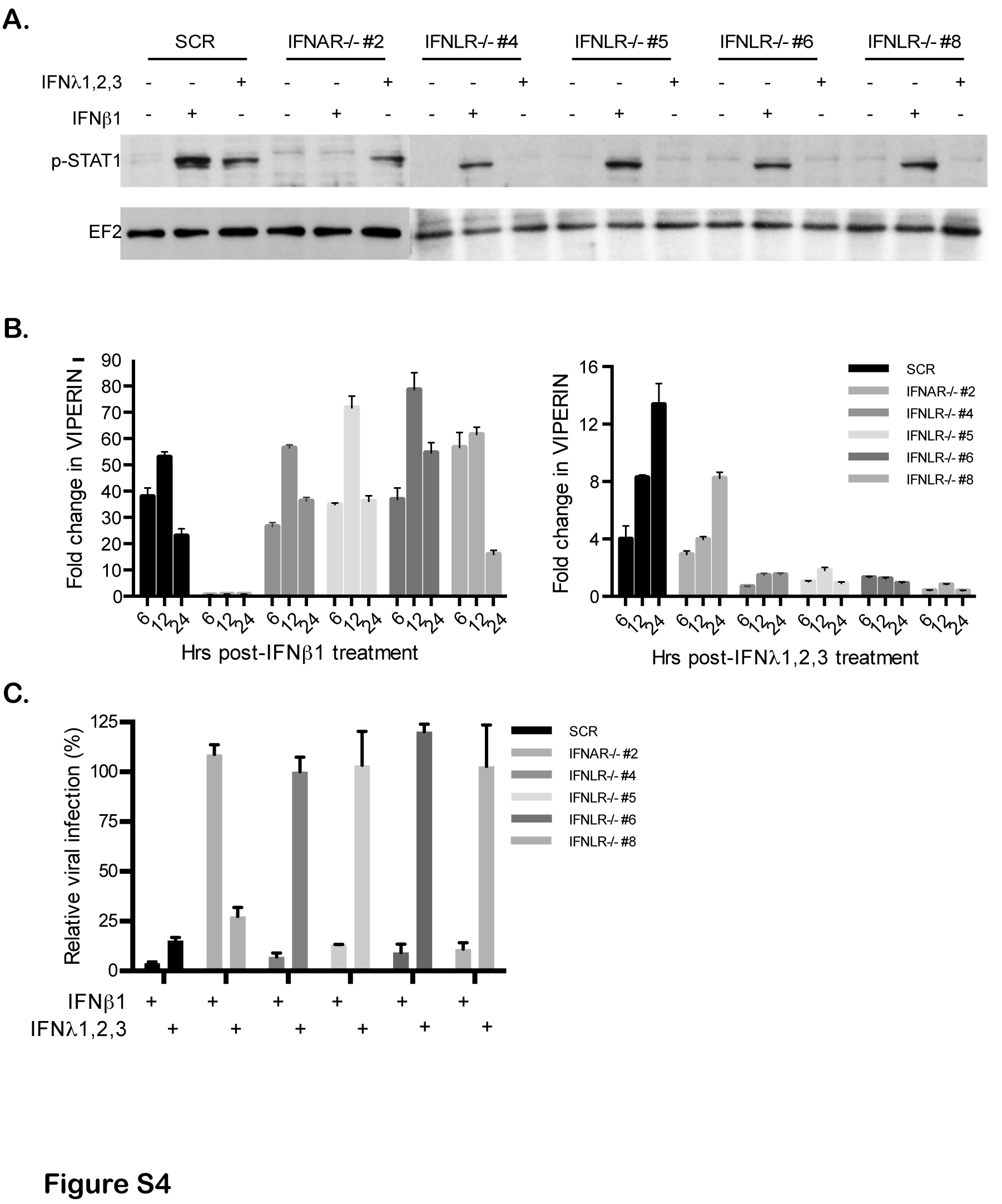

Supplement: Figure S4 — Characterization of different T84 IFNAR and IFNLR knockout (KO) cell clones generated using the CRISPR/Cas system. (A) IFNAR and IFNLR KO clones were treated with type I interferons (IFN) (β) (2,000 RU/mL equivalent 8 ng/mL) or type III IFN (λ1−3) (300 ng/mL) for 1 h and IFN signaling was measured by immunoblotting for pSTAT1 Y701. EF-2 is used as a loading control. A representative immunoblot out of three independent experiments is shown. (B) Same as (A), except that IFN signaling was evaluated by monitoring induction of IFN-stimulated genes by relative quantification of Viperin at indicated times post-IFN treatment using qRT-PCR. Data are normalized to TBP and HPRT1 and are expressed relative to untreated control cells of each time point. A representative experiment, out of three independent experiments is shown. (C) T84 cell lines were treated with type I IFN (β) (2,000 RU/mL equivalent 8 ng/mL) or type III IFN (λ1−3) (300 ng/mL) for 2 h prior to infection with VSV-expressing luciferase (multiplicity of infection = 1) and viral replication was assayed by measuring the luciferase activity. Results are expressed relative to mock-IFN-treated control cells generated with a scrambled control gRNA (set to 100). The mean value obtained from two independent experiments is plotted. Error bars indicate the SD. [file Image_4.TIF]

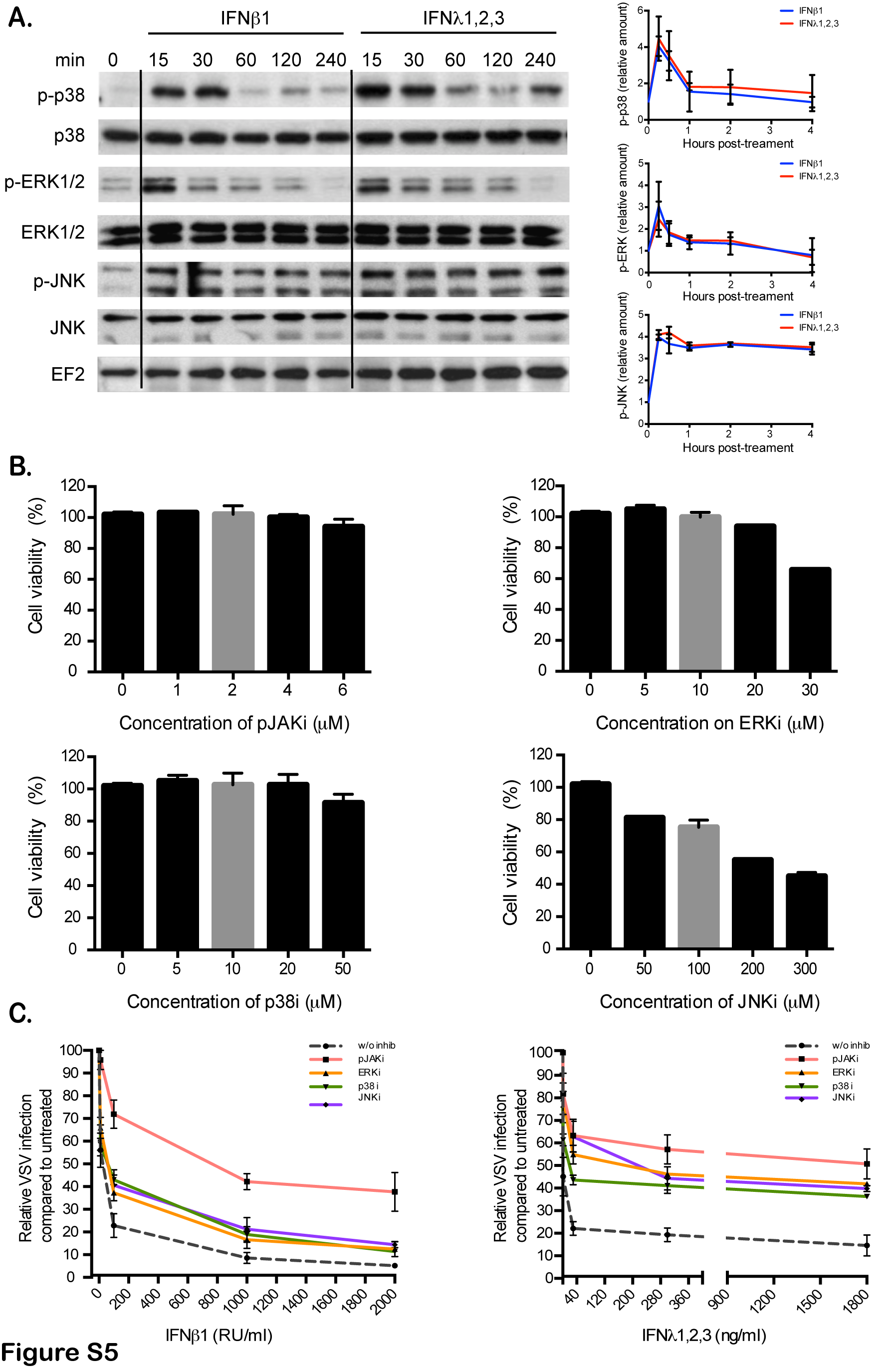

Supplement: Figure S5 — Role of MAP kinase pathway in type I and III interferon (IFN) antiviral activity. (A) T84 cells were treated with type I IFN (β) (2,000 RU/mL equivalent 8 ng/mL) or type III IFN (λ1−3) (300 ng/mL) for the indicated time points. The levels of phosphorylation of MAPkinases p38, ERK, and JNK were assessed by Western blot analysis. p38, ERK, JNK, and EF-2 were used as loading control. The phosphorylation of the MAP kinases was quantified and expressed relative to untreated cells (right panel). Data represent the mean values of three independent experiments. (B) T84 cells were treated with increasing concentrations of JAK and MAP kinase inhibitors. 24 h posttreatment of inhibitors the cell viability was assessed by MTT assay in triplicates. (C) T84 cells were pre-incubated for 30 min with 2 μM Pyridone 6 (pan-JAK inhibitor), 10 μM U0126 (ERK inhibitor), 10 μM SB202190 (p38 inhibitor) and 100 μM SP600125 (JNK inhibitor). Then the indicative concentrations of type I or III IFN were added in parallel to the inhibitor. Two hours post-IFN treatment cells were infected with VSV expressing luciferase (multiplicity of infection = 1) and viral replication was assayed by measuring the luciferase activity. Data were normalized to in no IFN-treated samples for each inhibitor. The mean value obtained from three independent experiments is shown. Error bars indicate the SD. [file Image_5.TIF]

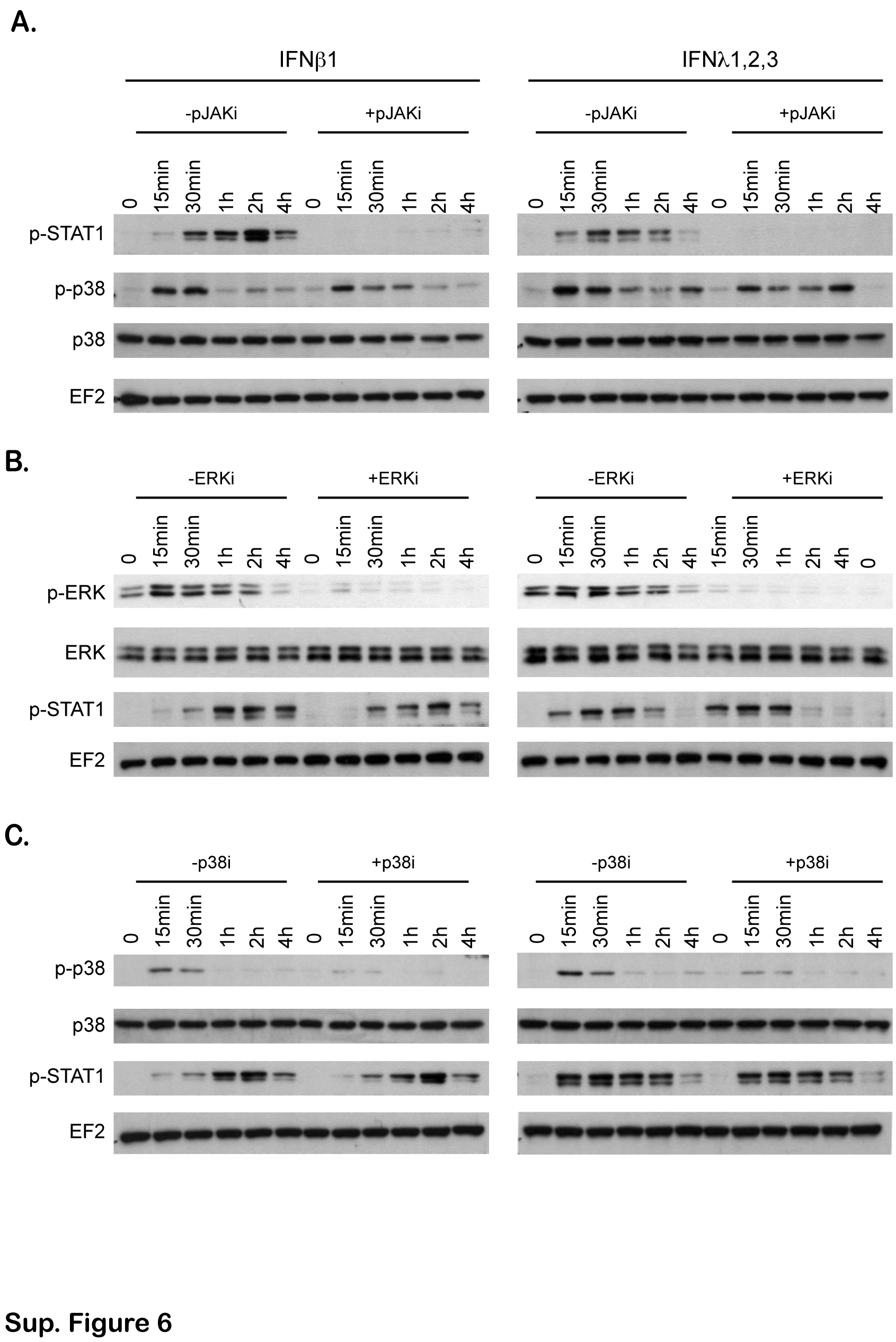

Supplement: Figure S6 — Specific inhibition of MAP kinases phosphorylation. T84 intestinal epithelial cells were pre-treated with JAK and MAPK inhibitors for 30 min prior to interferon treatment. Cells were harvested at different times posttreatment and the extent of JAK or MAPK inhibition was addressed by Western blot analysis. The specificity of each inhibitor was controlled by monitoring the phosphorylation status of JAK and all MAPKs. (A) 2 μM Pyridone 6 (pan-JAK inhibitor). (B) 10 μM U0126 (ERK inhibitor). (C) 10 μM SB202190 (p38 inhibitor). [file Image_6.TIF]
